# Mycobacterial Acid Tolerance Enables Phagolysosomal Survival and Establishment of Tuberculous Infection In Vivo

**DOI:** 10.1016/j.chom.2016.07.007

**Published:** 2016-08-10

**Authors:** Steven Levitte, Kristin N. Adams, Russell D. Berg, Christine L. Cosma, Kevin B. Urdahl, Lalita Ramakrishnan

**Affiliations:** 1 Molecular Immunity Unit, Department of Medicine, University of Cambridge, MRC Laboratory of Molecular Biology, Cambridge CB2 0QH, UK; 2 Molecular and Cellular Biology Graduate Program and Medical Scientist Training Program, University of Washington, Seattle, USA; 3 Center for Infectious Disease Research, Seattle, WA 98195, USA; 4 Department of Pediatrics, University of Washington, Seattle, WA 98109, USA; 5 Department of Microbiology, University of Washington, Seattle, WA 98109, USA; 6 Department of Immunology, University of Washington, Seattle, WA 98109, USA

## Abstract

The blockade of phagolysosomal fusion is considered a critical mycobacterial strategy to survive in macrophages. However, viable mycobacteria have been observed in phagolysosomes during infection of cultured macrophages, and mycobacteria have the virulence determinant MarP, which confers acid resistance in vitro. Here we show in mice and zebrafish that innate macrophages overcome mycobacterial lysosomal avoidance strategies to rapidly deliver a substantial proportion of infecting bacteria to phagolysosomes. Exploiting the optical transparency of the zebrafish, we tracked the fates of individual mycobacteria delivered to phagosomes versus phagolysosomes and discovered that bacteria survive and grow in phagolysosomes, though growth is slower. MarP is required specifically for phagolysosomal survival, making it an important determinant for the establishment of mycobacterial infection in their hosts. Our work suggests that if pathogenic mycobacteria fail to prevent lysosomal trafficking, they tolerate the resulting acidic environment of the phagolysosome to establish infection.

## Highlights

• In vivo, newly infecting mycobacteria are rapidly trafficked to lysosomes within macrophages
• The mycobacterial acid tolerance determinant MarP enables lysosomal survival and growth
• Phagolysosomal mycobacteria can successfully establish infection, which is MarP dependent

## Graphical Abstract

**Figure:**
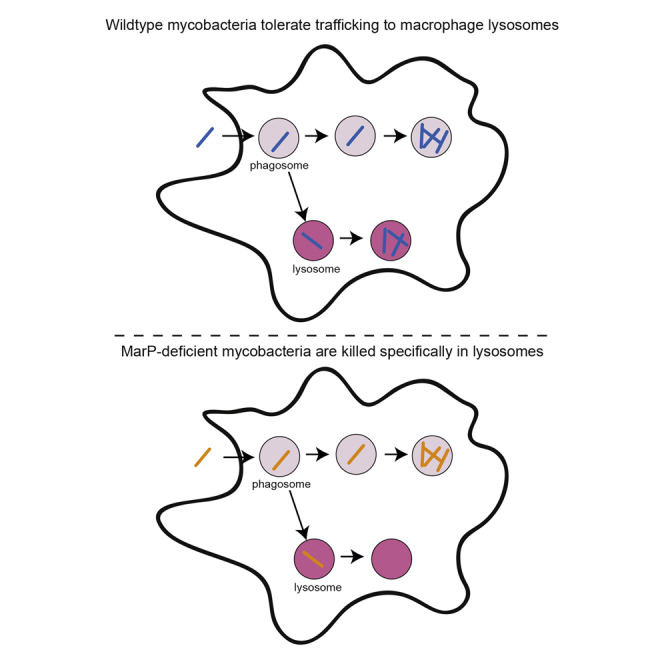

## Teaser

Blocking phagolysosomal fusion is considered critical for mycobacterial survival within macrophages. Levitte et al. (2016) show that while a substantial proportion of macrophage infecting mycobacteria are trafficked to lysosomes in vivo, the acid tolerance determinant MarP enables them to survive and replicate within lysosomes, thereby enhancing their ability to establish infection.

## Introduction

As first-line immune defense cells, macrophages phagocytose invading microbes, delivering them to lysosomes for degradation (Huynh and Grinstein, 2007). Therefore, to survive intracellularly, pathogens must avoid phagosomal fusion with lysosomes, survive within lysosomal compartments, or escape out of the phagosome to reside in the cytosol (Asrat et al., 2014). Studies in cultured macrophages have found that mycobacteria are capable of occupying all these subcellular niches (Cambier et al., 2014a).

Active avoidance of phagosome-lysosome fusion has been noted as a significant mycobacterial survival strategy, particularly during the innate immune phase of infection (Asrat et al., 2014, Behar and Baehrecke, 2015, Kasper et al., 2015, MacMicking, 2008, Tan and Russell, 2015). The idea that phagosomal blockade is integral to *Mycobacterium*’s intracellular survival and growth has been bolstered by findings that pretreatment of macrophages with gamma-interferon (IFNγ), a cytokine produced predominantly during the adaptive immune response, increases phagolysosomal fusion and decreases bacterial survival (Schaible et al., 1998). These findings led to the conclusion that IFNγ increases macrophage microbicidal capacity by enhancing mycobacterial trafficking to lysosomes (see Table S1 available online) (Flynn and Chan, 2001, Schaible et al., 1998, Via et al., 1998). However, IFNγ enhances macrophage killing through multiple mechanisms (Nunes-Alves et al., 2014), and dead mycobacteria are trafficked to macrophage lysosomes independently of IFNγ (Armstrong and Hart, 1975, Barker et al., 1997). Therefore, the enhanced killing mediated by IFNγ might be the cause of increased lysosomal trafficking rather than the effect.

Furthermore, three lines of evidence suggest that mycobacteria can tolerate lysosomal trafficking: (1) multiple studies find a substantial proportion of infecting *M. tuberculosis* (Mtb) in phagolysosomes soon after infection of cultured macrophages, with 10%–25% at 2–3 hr and 15%–36% at 24 hr (Table S1); (2) Mtb survives and even replicates upon delivery into lysosomes, either through Fc receptor-mediated phagocytosis or by co-infection with the lysosomal pathogen *Coxiella burnetti* (Armstrong and Hart, 1975, Gomes et al., 1999); and (3) the membrane serine protease Rv3671c (MarP), which was identified in an in vitro screen for acid tolerance determinants, is also required for virulence (Small et al., 2013, Vandal et al., 2008), consistent with the idea that Mtb experiences acid stress in vivo.

The macrophage lysosomal avoidance and tolerance strategies employed by mycobacteria have been described for cultured macrophages (Table S1). Here, we directly address in vivo the prevalence and consequences of lysosomal trafficking using the optically transparent zebrafish larva, which allows for real-time tracking of infection with *M. marinum* (Mm), a close genetic relative of Mtb and a natural agent of tuberculosis (TB) in ectotherms (Cambier et al., 2014a). We find that innate macrophages, in the absence of IFNγ stimulation, deliver a substantial proportion of infecting mycobacteria to lysosomes. However, this potentially host-beneficial innate immune strategy is effectively counteracted by mycobacterial MarP, which specifically mediates bacterial survival and growth within lysosomes.

## Results

### Mtb Resides in Macrophage Phagosomes and Phagolysosomes after Aerosol Infection of Mice

**Figure 1:**
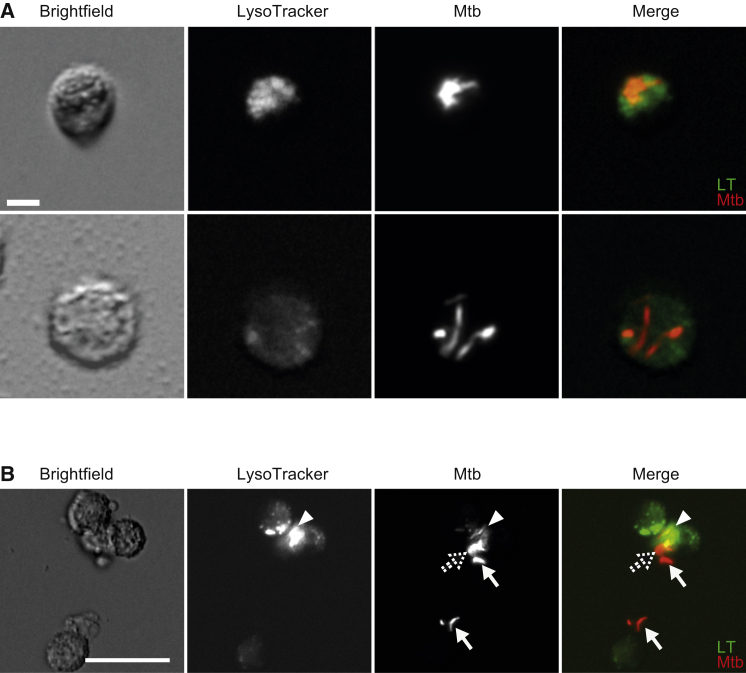
Direct Ex Vivo Phagolysosomal Localization of *M. tuberculosis* Images of red fluorescent Mtb H37Rv from infected lung. Cells were sorted from lung tissue at 13 days post-infection (dpi) (A) or 19 dpi (B) and stained with LysoTracker (LT) Green dye. (A) The top images show an infected macrophage in which the Mtb colocalizes with LT, while the bottom images show an infected macrophage in which the Mtb does not colocalize with LT. Representative of two mice. Scale bar, 5 μm. (B) Arrowheads depict bacteria that colocalize with LT, while arrows depict bacteria that do not colocalize with dye. The dotted arrow marks partially colocalized bacteria. Representative of three mice. Scale bar, 25 μm.

Macrophages derived from a variety of hosts rapidly traffic a proportion of infecting mycobacteria to lysosomes in vitro (Table S1). We asked whether this was also the case in vivo during the first macrophage-mycobacterium interaction, which in humans and mice is thought to occur within lung-resident macrophages (Verrall et al., 2014). We infected mice with ∼200 fluorescent Mtb by aerosolization and assessed lysosomal trafficking as judged by bacterial co-localization with LysoTracker, an acidophilic dye that labels lysosomes. We chose 13 and 19 days post-infection, time points that flank the 14–15 day time point when IFNγ-producing T cells begin to arrive in the lung after aerosol Mtb infection (Khader et al., 2007). (Analysis before 13 days was precluded by the rarity of Mtb-infected cells.) Mtb in lysosomal compartments was readily observed in all animals (two at 13 days and three at 19 days) (Figure 1), though the relative rarity of infected cells even at these time points precluded quantification of the extent of lysosomal trafficking. Because lung-resident macrophages are the predominant infected cell type early after aerosolized Mtb infection (Urdahl, 2014, Wolf et al., 2007), our findings suggest that these first-responding cells can traffic Mtb to lysosomes in the sole context of innate immunity. A human study of HIV-positive TB patients also found ∼30% of Mtb in phagolysosomes of alveolar macrophages obtained by bronchoalveolar lavage, although the duration of infection was unknown in this case (Table S1) (Mwandumba et al., 2004).

### A Proportion of Mm Is Rapidly Trafficked to Lysosomes after Infection of Larval Zebrafish

**Figure 2:**
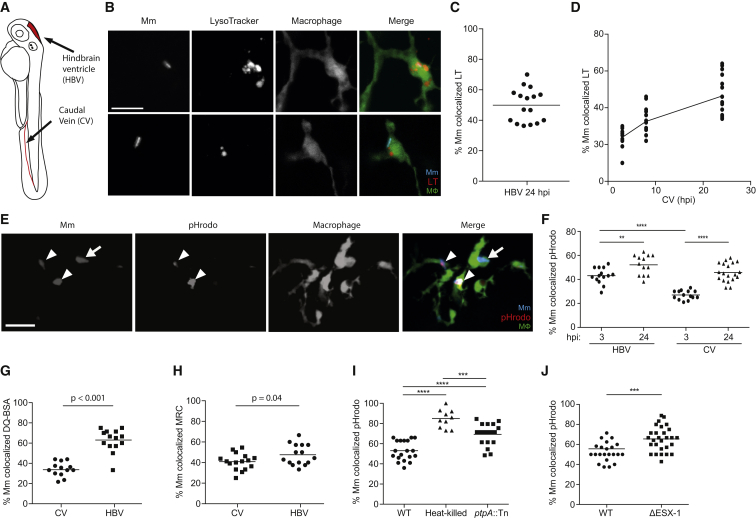
In Vivo Phagolysosomal Trafficking of *M. marinum* (A) Illustration of zebrafish larva with injection sites outlined in red. The hindbrain ventricle (HBV) is accessible to recruited myeloid cells, while the caudal vein (CV) traverses the caudal hematopoietic tissue where myeloid cells develop. (B) Confocal images of blue fluorescent Mm that have been phagocytosed by green fluorescent macrophages in the brain of a 2 day post-fertilization (dpf) larva stained with red LT; one bacterium shown colocalizes with LT, and the other does not. Scale bar, 10 μm. (C) Percent of Mm colocalizing with LT dye 24 hr post infection (hpf) into the HBV, representative of three experiments. (D) Percent of Mm colocalizing with LT dye at 3, 8, and 24 hpi in the CV, representative of three experiments. (E) Confocal images of blue fluorescent Mm that were pre-labeled with red pHrodo prior to infection into the HBV of 2dpf larvae with green fluorescent macrophages; bacteria that colocalize with pHrodo (arrowheads) and one that does not (arrow). Scale bar, 10 μm. (F) Percent of Mm colocalizing with pHrodo at 3 and 24 hpi in the HBV or CV of a 3 dpf larva, representative of two experiments. (G and H) Percent of Mm colocalizing with DQ-BSA (G) or MR-Cathepsin (MRC) (H) imaged at 24 hpi following infection in the HBV or CV of 2 dpf larvae, representative of two experiments each. (I) Percent of live, heat-killed, or ptpA::Tn Mm pre-labeled with pHrodo prior to infection into the HBV of 2 dpf larvae imaged at 24 hpi, representative of three experiments. (J) Percent of ΔESX-1 Mm colocalizing with pHrodo imaged at 24 hpi in the HBV of 2 dpf larvae, representative of three experiments. Significance tested using one-way ANOVA with Tukey's post-test (F and I) or two-tailed unpaired t test (G, H, and J). Each point in (C), (D), and (F)–(J) represents one larva, with mean depicted as a horizontal line. See also Figures S1 and S2.

To probe lysosomal trafficking of mycobacteria in vivo and its consequences in real time, we turned to zebrafish larvae infected with Mm. In cultured macrophages, a similar proportion of Mm (21% at 4 hr post-infection [hpi]) as Mtb is rapidly trafficked to lysosomes (Table S1) (Barker et al., 1997). We infected zebrafish larvae with fluorescent Mm in the hindbrain ventricle (HBV), an epithelium-lined cavity to which myeloid cells are rapidly recruited in response to infecting Mm (Cambier et al., 2014b) (Figure 2A). Using LysoTracker, which has been shown to label lysosomes in larval zebrafish (Peri and Nüsslein-Volhard, 2008), we found that a substantial proportion of the bacteria was in acidified compartments within 24 hpi (Figures 2B and 2C). Similar results were obtained after injecting bacteria into the caudal vein (CV), which traverses the hematopoietic tissue, the site of intermediate myelopoiesis giving rise to circulating monocytes (Clements and Traver, 2013) (Figure 2A); mycobacteria were rapidly and progressively delivered to acidified compartments of circulating myeloid cells within 3 to 24 hpi (Figure 2D). In vitro, the mycobacterial phagosome has been reported to be only slightly acidified to a pH of 6.2 (Tan and Russell, 2015), so we asked whether the bacteria colocalizing with LysoTracker were in slightly acidified phagosomes or in bona fide lysosomal compartments, which have a pH <5 and contain hydrolytic enzymes. We directly assessed the pH at the bacterial surface by labeling Mm prior to infection with pHrodo, a pH-sensitive dye. Incubation of pHrodo-stained Mm in phosphate-citrate buffer at a range of pH values showed that fluorescence was greatly increased in bacteria experiencing a pH of ≤ 5.0 (Figure S1A). We used a fluorescence intensity cutoff for imaging pHrodo-stained bacteria in the zebrafish so as to exclude those experiencing pH >5.0 (Figure S1A). pHrodo-stained bacteria were detected using this cutoff soon after both HBV and CV infection (Figures 2E and 2F), and co-staining with LysoTracker and pHrodo showed strong overlap (Figures S1B and S1C). Staining with MR-Cathepsin and DQ-BSA, fluorogenic protease substrates that fluoresce only upon hydrolysis and label macrophage lysosomes in zebrafish (Peri and Nüsslein-Volhard, 2008), revealed a similar proportion of phagolysosome-localized bacteria as pHrodo staining (Figures 2G and 2H). Thus, during in vivo infection, macrophages rapidly deliver a proportion of mycobacteria to bona fide lysosomal compartments characterized by hydrolase activity and pH <5.

In the context of cultured macrophages, the observed distribution of Mtb and Mm in phagosomes versus phagolysosomes is the result of counteracting macrophage and bacterial strategies. While the macrophage can consign a significant proportion to the lysosome, the majority of bacteria actively avoid this fate, as evidenced by the finding that killed bacteria are rapidly trafficked to lysosomes (Armstrong and Hart, 1971, Barker et al., 1997). Multiple determinants have been implicated in Mtb’s avoidance of lysosomes, e.g., the tyrosine phosphatase PtpA (Bach et al., 2008), and the specialized secretion system ESX-1 (MacGurn and Cox, 2007). To further validate our findings in zebrafish macrophages, we first asked if Mm actively avoids lysosomal trafficking by comparing pHrodo-stained live and heat-killed bacteria (Figure 2I). We observed increased lysosomal localization of killed Mm. Moreover, both a Mm *ptpA* (*mmar_3309*) transposon insertion mutant and an ESX-1-deficient mutant displayed increased lysosomal localization albeit less than heat-killed bacteria (Figures 2I and 2J), suggesting the presence of additional mycobacterial factors that mediate phagolysosomal blockade, as has been reported for Mtb (Asrat et al., 2014).

In sum, our findings suggest that Mm actively blocks phagosome-lysosome fusion during zebrafish infection through mechanisms similar to those used by Mtb in cultured macrophages. But similarly to observations in cultured macrophages, this blockade is only partially successful. Conversely, macrophages, regardless of species, are robustly equipped to consign a substantial proportion of infecting mycobacteria to lysosomal compartments.

### Trafficking of Mycobacteria to Lysosomes Does Not Require IFNγ

Our observed proportions of phagolysosomal bacteria in vivo at 24 hpi (45%–52%) were a little higher than the 15%–36% reported for Mtb and Mm in cultured macrophages (Figures 2C and 2D; Table S1). Since IFNγ stimulates phagosome-lysosome fusion in cultured macrophages (Schaible et al., 1998, Via et al., 1998), we asked if it was responsible for the small increase in lysosomal trafficking observed in vivo. In mammals, IFNγ is predominantly produced by T lymphocytes, which have not yet developed in the zebrafish larvae (Clements and Traver, 2013); however, there are also innate sources of IFNγ (e.g., natural killer cells) (Renshaw and Trede, 2012). Both zebrafish IFNγ orthologs—*ifng1-1* and *ifng1-2*—were induced largely in a RAG-dependent fashion at 6 weeks post-infection in adult zebrafish. This result is similar to mouse Mtb infection, where adaptive immune cells are the predominant source of IFNγ (Baldridge et al., 2010, Flynn and Chan, 2001) (Figures S2A and S2B). Similar trends were observed by 2 weeks post-infection in adults (Figures S2C and S2D). To look for innate sources of IFNγ with a more sensitive assay, we tested *ifng1-2* expression in response to a potent IFNγ inducer, the TLR3 agonist poly(I:C) (Aggad et al., 2010) (Figure S2E). We confirmed its rapid induction (4 hr post-administration) in adult animals from both innate and adaptive immune sources, but predominantly the latter (Figure S2E). In the larva also, we observed poly(I:C)-mediated IFNγ induction starting at 4 days postfertilization but failed to detect induction of either homolog earlier in development (Figure S2F). This pattern held up for Mm infection: neither *ifng1-1* nor *ifng1-2* induction was detected before 4 days post-fertilization (2 days post-infection) (Figure S2G). In sum, our data suggest that zebrafish induce IFNγ in response to Mm similarly to mice responding to Mtb. While some IFNγ can be made by innate sources, these innate cells have yet to mature during the window used in our studies on phagolysosomal fusion. While we failed to observe induction of IFNγ at early time points, this does not exclude small amounts being present from early during development. We addressed the potential contribution of a small amount of IFNγ by assessing infection in larvae injected with *ifng1-1* and *ifng1-2* morpholinos, *crfb17* morpholino (part of the signaling machinery for both IFNγ1 and IFNγ2), and a mutant in *crfb17* (Aggad et al., 2010, Sieger et al., 2009). We did not observe hypersusceptibility to infection in any of these conditions (Figures S2H–S2J). In sum, this early phagolysosomal fusion likely represents the intrinsic ability of the macrophage to deliver infecting bacteria to lysosomal compartments.

### The Host-Protective Effect of Lysosomal Trafficking Is Limited through the Action of the Mycobacterial Serine Protease MarP

**Figure 3:**
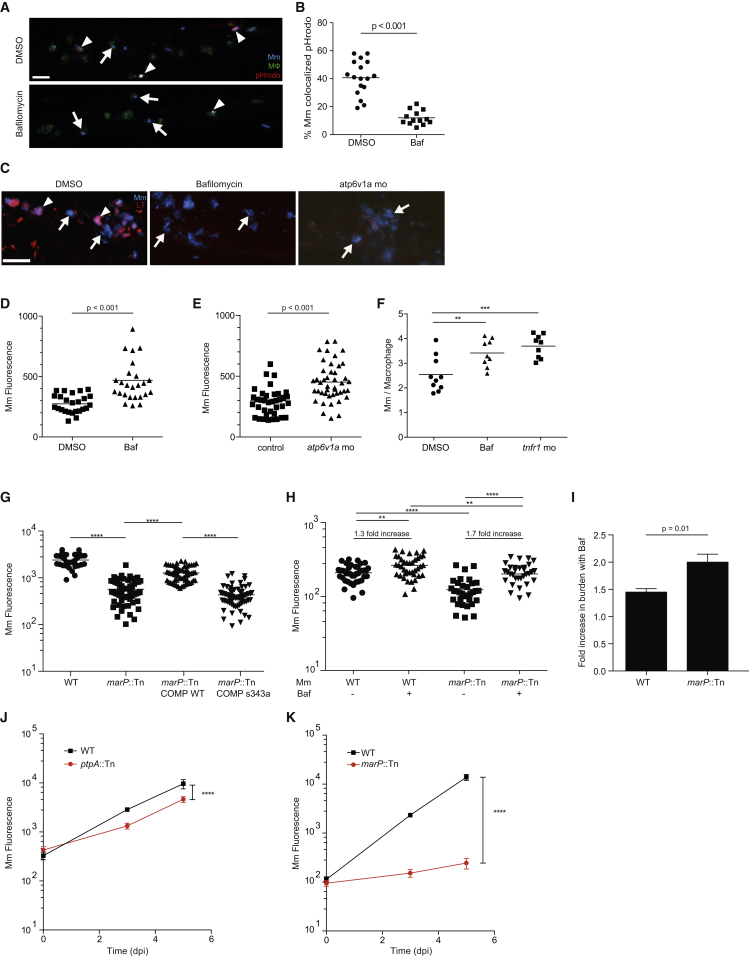
Lysosomal Trafficking Is a Host-Beneficial Process, which Is Counteracted by Bacterial MarP (A) Confocal images of 3 dpf larvae that were infected with Mm in the CV at 2 dpf and then treated for 24 hr with 50 nM Bafilomycin (Baf) or DMSO control. Arrowheads denote bacteria labeled with pHrodo, while arrows show pHrodo-negative bacteria. (B) Percent of Mm colocalizing with pHrodo at 24 hpi as shown in (A), representative of two experiments. (C) Confocal images of 3 dpf larvae that were infected in the CV with blue fluorescent Mm at 2 dpf and stained with red LT at 3 dpf following treatment with DMSO, 50 nM Baf, or morpholino targeting *atp6v1a* at 0 dpf. Arrowheads denote bacteria that colocalize with LT, while arrows denote LT-negative bacteria. (D) Bacterial burden (FPC) measured at 24 hpi with 300 Mm; fish were randomly assigned to DMSO or 50 nM Baf treatment immediately after infection, representative of three experiments. (E) Bacterial burden measured at 24 hpi with 300 Mm in larvae injected at 0 dpf with a morpholino targeting *atp6v1a* or control, representative of two experiments. (F) Average intramacrophage burden at 40 hpi with 60 Mm into the CV comparing larvae treated with DMSO or 50 nM Baf following infection, or injected with a morpholino targeting *tnfr1* at 0 dpf, which was used as a positive control, representative of three experiments. (G) Bacterial burden measured at 3 dpi following infection with wild-type Mm, *marP*::Tn, *marP*::Tn transformed with a plasmid containing Mtb *marP*, or *marP*::Tn transformed with a plasmid containing Mtb *marP* with a mutation in the active site serine (S343A), representative of two experiments. (H) Bacterial burden at 1 dpi following infection with wild-type or *marP*::Tn Mm and treatment with either DMSO or 50 nM Baf, representative of three experiments. The fold increase in burden following treatment with Baf is shown for wild-type and *marP*::Tn. (I) Fold increase in bacterial burden during infection with wild-type or *marP*::Tn Mm following treatment with 50nM Baf. Shown is the average of four experiments as in (H) ± SEM. (J) Bacterial burden measured at 0, 3, and 5 dpi following infection with 250 wild-type or *ptpA*::Tn Mm with mean and 95% CI shown, representative of three experiments. (K) Bacterial burden at 0, 3, and 5 dpi following infection with wild-type or *marP*::Tn Mm with mean and 95% CI shown, representative of three experiments. Significance tested using two-tailed unpaired t test (B, D, E, and I–K) or one-way ANOVA with Tukey’s post-test (F–H). Each point in (B) and (D)–(H) represents one larva. See also Figure S3.

Studies in cultured macrophages have come to different conclusions about the consequences of mycobacterial phagolysosomal trafficking depending on the stimulus used to achieve it. Mycobacteria delivered into phagolysosomes by opsonization or by co-infection with the lysosomally localized bacterium *Coxiella burnetti* survived and even replicated in lysosomes, whereas bacteria reaching lysosomes following IFNγ stimulation of the macrophages were killed (Armstrong and Hart, 1975, Cosma et al., 2003, Gomes et al., 1999). To test macrophage-intrinsic phagosomal maturation as a microbicidal effector mechanism in vivo, we disrupted phagosomal maturation by treating larvae with the vATPase inhibitor Bafilomycin (Peri and Nüsslein-Volhard, 2008), or used a translation-blocking morpholino targeting *atp6v1a*, a subunit required for vATPase function in the zebrafish (Horng et al., 2007). LysoTracker and pHrodo staining each confirmed disruption of lysosomal localization in the context of infection (Figures 3A–3C). Bafilomycin treatment and *atp6v1a* knockdown each increased bacterial burdens within 24 hr (Figures 3D and 3E). This increase in bacterial burdens was reflected in increased bacterial replication within individual larval macrophages (Figure 3F), similar to the reduced microbicidal capacity of TNF-deficient macrophages (Figure 3F) (Clay et al., 2008). Thus, phagosomal maturation restricts the progression of infection by decreasing intramacrophage replication of Mm.

The fact that Mm infection progresses overall despite growth restriction mediated by the lysosome suggested one of two explanations: either that the expansion of overall infection is driven exclusively by the bacterial population avoiding lysosomes, or that bacteria can replicate in both phagosomes and lysosomes. To distinguish between these possibilities, we used two mutants—a transposon mutant in the ortholog (*mmar_5159*) of the Mtb membrane serine protease MarP, which is required for acid tolerance in vitro and virulence in mice (Vandal et al., 2008), and *ptpA*::Tn, which displays increased lysosomal trafficking (Figure 2I). We confirmed that *marP*::Tn was acid sensitive and hypersusceptible to lipophilic antibiotics in vitro, and attenuated in vivo (Figures S3A–S3D and Figure 3G), consistent with the phenotypes of its Mtb counterpart (Vandal et al., 2008). Because of MarP’s multiple in vitro phenotypes (increased susceptibility to acid, hydrophobic antibiotics and detergents, reactive oxygen species, and nitric oxide), it has not been clear which of these is responsible for its attenuation in vivo (Ehrt et al., 2015, Stallings and Glickman, 2010). We reasoned that if defective lysosomal tolerance contributes to *marP*::Tn attenuation, then Bafilomycin should enhance *marP*::Tn growth more than wild-type. It did (2.0- ± 0.15-fold increase in burden for *marP*::Tn versus 1.5- ± 0.06-fold for wild-type, p = 0.01) (Figures 3H and 3I). Furthermore, *marP*::Tn was ∼25 times more attenuated than *ptpA*::Tn during a 5-day infection (Figures 3J and 3K), suggesting that, in vivo, tolerance of the acidic lysosomal environment is a more significant determinant of mycobacterial growth than avoidance of phagosome-lysosome fusion. This is consistent with findings in the mouse-Mtb aerosol infection model where the MarP mutant is severely attenuated but the PtpA mutant is not (Grundner et al., 2008, Vandal et al., 2008). In sum, lysosomal trafficking is a host-beneficial strategy that limits intramacrophage mycobacterial growth and thereby expansion of infection in the granuloma. The effectiveness of this strategy is substantially offset, however, by MarP-mediated tolerance of lysosomal trafficking.

### Mycobacteria Delivered to Phagolysosomes Can Successfully Establish Infection in a MarP-Dependent Fashion

**Figure 4:**
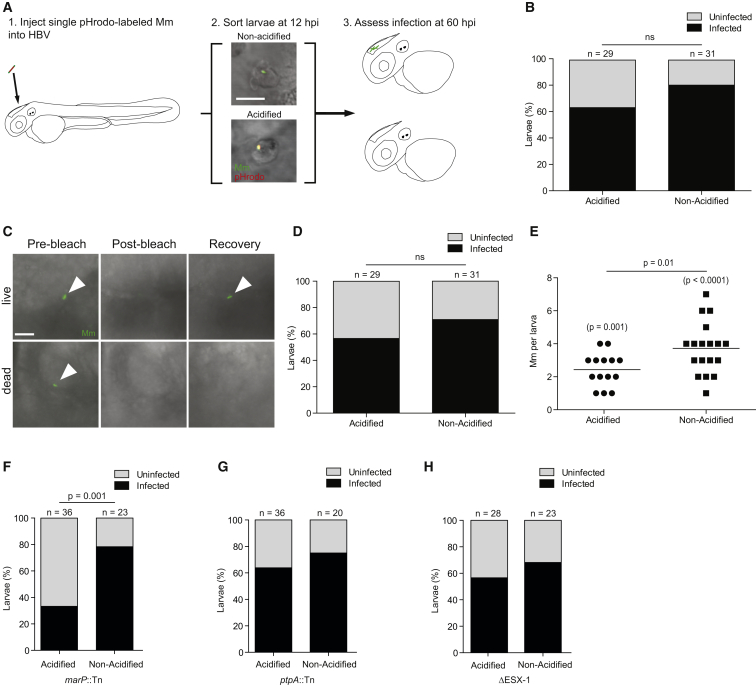
Lysosomal Trafficking Fails to Eradicate Mm Infection (A) Cartoon diagram of infectivity experiment. Scale bar, 10 μm. (B) Percent of zebrafish larvae still infected at 60 hpi following infection in the HBV with single pHrodo-labeled Mm and sorting into acidified/non-acidified groups, representative of three experiments. (C) Photobleaching assay to discern live from killed bacteria at the end of the infectivity assay showing a live bacterium that recovers fluorescence and a dead one that does not. Scale bar, 5 μm. (D) Data in (B) amended so that larvae with only non-recovering bacteria are placed into the uninfected category, representative of three experiments. (E) Bacterial numbers at the end of the infectivity experiment, showing only bacteria that recovered after photobleaching, representative of three experiments. p values in parentheses reflect the statistical significance of comparing each larva in that group to the starting bacterial number (1 in each fish) in a Wilcoxon matched-pair signed rank test. (F–H) Percentage of zebrafish larvae still infected at 60 hpi which contained pHrodo-labeled *marP*::Tn (F), *ptpA*::Tn (G), and ΔESX-1 (H) Mm separated by whether the bacteria were pHrodo-positive (lysosomal) or pHrodo-negative (phagosomal) at sorting, representative of two (ΔESX-1) or three (*marP*::Tn, *ptpA*::Tn) experiments. Significance tested using two-tailed unpaired t test (E) or Fisher’s exact test (B, D, and F–H). Each point in (E) represents one larva. See also Figure S4.

We probed the basis of lysosomal trafficking as a host-protective mechanism (bactericidal, bacteriostatic, or simply slowing bacterial growth) and, conversely, how mycobacterial MarP counters this strategy. The optical transparency of the zebrafish, particularly its HBV, allowed us to map the fate of individual mycobacteria following initial distribution into phagosomes versus phagolysosomes. We injected single pHrodo-labeled bacteria into the HBV (Figure 4A), sorted the animals based on whether the bacteria were found within pHrodo-labeled phagolysosomes at 12 hpi, and then tracked bacterial fates for 48 hr to determine if the host was still infected based on the presence of fluorescent Mm in the HBV (Figure 4A). The fraction of infected larvae at 60 hpi was not significantly different between the phagosomal and phagolysosomal groups (Figure 4B). We confirmed that the bacteria were alive and metabolically active by photobleaching them and assessing for recovery of fluorescence (Figures 4C and 4D). In infected animals, we assessed the extent of bacterial growth in phagosomes versus phagolysosomes by enumerating the number of bacteria at 60 hpi. Mm replicated within the phagolysosome, though at a slower rate than in the non-acidified phagosome (Figure 4E).

Consistent with a primary role of MarP in tolerating the lysosomal environment, *marP*::Tn was cleared more than wild-type only when it was trafficked to the phagolysosome (Figure 4F). Individual mutant bacteria that were in non-acidified phagosomes survived similarly to wild-type (compare Figures 4F and 4D). In contrast, *ptpA*::Tn and ΔESX-1, which are preferentially trafficked to the phagolysosome but are not sensitive to acid in vitro, survived in either compartment similarly to wild-type (Figures 4G and 4H).

In sum, these experiments suggest that lysosomal residence is fully conducive to mycobacterial survival and also supports growth, albeit at a slower rate than nonacidified phagosomes. Mycobacterial MarP mediates this lysosomal survival.

## Discussion

Our in vivo work detailing the progression of mycobacterial trafficking in macrophages and its consequences suggests that pathogenic mycobacteria encounter and counter macrophage phagolysosomal fusion from the earliest stage of infection. The lysosomal avoidance and tolerance strategies utilized by mycobacteria are not mutually exclusive, and our findings suggest that newly arriving mycobacteria employ a tiered strategy to successfully establish infection in first-responder macrophages. In the face of frequently successful phagosome-lysosome fusion by these macrophages, mycobacteria must fall back on their ability to contend with the microbicidal arsenal of the lysosome (Vandal et al., 2009). The increased lysosomal localization (40%–50%) we observe in vivo over cultured macrophages (15%–36%) may reflect the enhancement of intrinsic macrophage phagolysosomal fusion capacity by cues in vivo which must derive from innate immunity, since they are independent of both IFNγ and Fc receptor (i.e., antibodies).

In terms of the consequences of phagolysosomal fusion, we find that it does not enhance the macrophage’s ability to kill bacteria, nor does it induce bacteriostasis. Mycobacteria continue to grow in both compartments, albeit more slowly in phagolysosomes. These in vivo findings differ from those in cultured macrophages, where lysosomal fusion was induced by Fc receptor-dependent phagocytosis and where decreased growth of the lysosomal bacteria was not observed (Armstrong and Hart, 1975). Our findings also differ from prior findings in vitro that while mycobacteria can survive transiently at low pH, they fail to replicate under these conditions (Asrat et al., 2014, Behar and Baehrecke, 2015, Kasper et al., 2015, MacMicking, 2008, Tan and Russell, 2015). The ability of in vivo macrophage phagolysosomes to limit mycobacterial growth to some extent may reflect the presence of lysosomally activated immune determinants such as nitric oxide (Vandal et al., 2009). This in turn suggests that, ultimately, lysosomal trafficking may serve as a host-protective mechanism. However, its efficacy may be limited later in infection, as supported by previous work showing that morphologically intact Mm were present in frog granuloma macrophage lysosomes in unchanging and substantial numbers throughout a 17–52 week observation period when overall infection burdens were increasing (Bouley et al., 2001).

Prior work has shown that MarP is a virulence determinant in the mouse model of TB and that it is required for acid tolerance in vitro (Vandal et al., 2008). However, the link between MarP’s role in acid tolerance and the attenuation of the *marP* mutant has remained tenuous because it has not been clear whether the mutant’s attenuation is due to its acid intolerance or due to other effects such as sensitivity to oxidative stress or to cell wall insults (Ehrt et al., 2015, Stallings and Glickman, 2010). Our work firmly links the acid tolerance conferred by mycobacterial MarP to its function as a virulence determinant during the early stages of infection. Importantly, we show that MarP specifically enables the establishment of infection by mycobacteria that have been consigned to lysosomes. This likely represents a critical determinant of the evolutionary survival of Mtb; human TB is thought to begin with the deposition of one to three bacteria into the lung alveolus (Bates et al., 1965, Cambier et al., 2014a), and the initial interaction between these bacteria and the host macrophage is thought to determine whether the infection is cleared or progresses (Verrall et al., 2014). MarP is widely conserved across mycobacterial species, which have been reported to survive (and even be enriched) in environments as hostile as volcanic rock at pH 1 (Walker et al., 2005) (Figure S4). Thus, acid tolerance likely evolved as a survival mechanism well before mycobacteria encountered macrophages or free-living amebae and joins the catalog of determinants for environmental survival that have been repurposed for host survival by pathogenic mycobacteria (Cambier et al., 2014a).

## Experimental Procedures

### Bacterial Strains and Methods

Wild-type Mm (strain M, ATCC #BAA-535) expressing tdTomato or mWasabi under the constitutive promoter *msp12* was used for measurement of total and intracellular bacterial burdens. *mmar_5159* and *mmar_3309* mutants were isolated from a library of Mm transposon mutants (C.L.C., unpublished data). See also Supplemental Experimental Procedures.

### Mouse Husbandry and Mtb Infection

Mouse husbandry and experiments were conducted in accordance with an animal study proposal approved by the Center for Infectious Disease Research Animal Care and Use Committee. C57BL/6 mice purchased from Jackson Laboratories or maintained in house were infected with ∼200 CFU of aerosolized Mtb strain H37Rv expressing mCherry in a Glas-Col infection chamber (Glas-Col, Terre Haute, IN). Two mice from each infection were sacrificed and lung homogenates plated to determine the deposition of Mtb.

### Zebrafish Husbandry and Mm Infections in Zebrafish

Zebrafish husbandry and experiments were in compliance with guidelines from the UK Home Office and the U.S. National Institutes of Health and approved by the University of Washington Institutional Animal Care and Use Committee. Transgenic lines were maintained as outcrosses to AB. Larvae were infected at 48–72 hr post-fertilization via CV or HBV injection using thawed single-cell suspensions with 75–200 bacteria delivered during infection unless noted otherwise. Fish containing single bacteria were identified 3 hpi by confocal microscopy and were again scored at 12 hpi for pHrodo sorting using cutoff parameters as in Figure S2A. See also Supplemental Experimental Procedures.

### Statistical Analyses and Image Analysis

Images were analyzed using Imaris 7.7–8.2 (Bitplane). The Venn diagram was generated using the Pan-Omics Research Venn Diagram Plotter (http://omics.pnl.gov). Statistical analyses were performed using Prism 6 (GraphPad) (not significant [ns], p ≥ 0.05; ^∗^ p < 0.05; ^∗∗^ p < 0.01; ^∗∗∗^ p < 0.001; ^∗∗∗∗^ p < 0.0001).

## Author Contributions

S.L., K.N.A., R.D.B., K.B.U., and L.R. conceived and designed experiments and analyzed the data. C.L.C. generated Mm mutants. S.L. and L.R. wrote the paper with input from C.L.C. and R.D.B. The figures were prepared by S.L.

## Supplemental Information

Document S1. Figures S1–S4, Table S1, and Supplemental Experimental Procedures

Document S2. Article plus Supplemental Information
