# Supplementary material for: Mycobacterial Acid Tolerance Enables Phagolysosomal Survival and Establishment of Tuberculous Infection In Vivo
Source: Cell Host Microbe. 2016 Aug 10;20(2):250–8. doi: 10.1016/j.chom.2016.07.007 (PMC4985559; doi:10.1016/j.chom.2016.07.007)
Supplement: Document S1. Figures S1–S4, Table S1, and Supplemental Experimental Procedures [file mmc1.pdf]

Cell Host & Microbe, Volume *20*

Supplemental Information

Mycobacterial Acid Tolerance Enables Phagolysosomal Survival and Establishment of Tuberculous Infection In Vivo

Steven Levitte, Kristin N. Adams, Russell D. Berg, Christine L. Cosma, Kevin B. Urdahl, and Lalita Ramakrishnan

Supplemental Figures and Tables

Figure S1

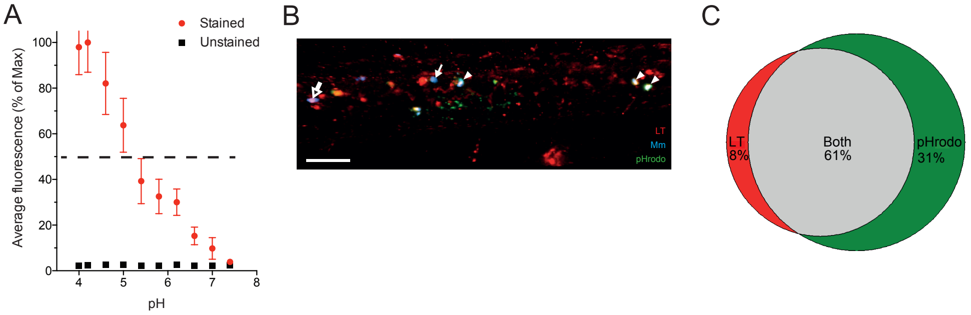

Figure S1. pHrodo efficiently labels acidified *M. marinum* (Mm), related to Figure 2. (A) Intensity of pHrodo fluorescence in stained Mm at various pH levels. Bacteria were pre-labeled with pHrodo, and then incubated in phosphate-citrate buffer at the indicated pH. The stained bacteria and unstained controls were imaged using the same confocal parameters as those used in zebrafish larvae. 3D surfaces were created from these images and pHrodo intensity was measured within each bacterium and intensity was plotted as a percentage of the maximum average value (pH 4.2). Shown is the average of at least 300 individual bacteria +/- one standard deviation. The dotted line represents the intensity cutoff used to assess pHrodo in vivo. (B) Confocal image of larva infected in the CV with pHrodo-labeled Mm, then stained with LysoTracker at 24hpi. White arrowheads denote bacteria that label with both LysoTracker and pHrodo, white arrow denotes a bacterium that does not label with either marker, and black arrow denotes a bacterium that labels with LysoTracker but not with pHrodo. Scale bar, 50µm. (C) Quantitative Venn diagram showing the distribution of Mm that were positive for either LysoTracker (LT), pHrodo or both in larvae infected in the CV with pHrodo-labeled Mm and then stained with LysoTracker at 24hpi with percentages noted, representative of two experiments.

Figure S2

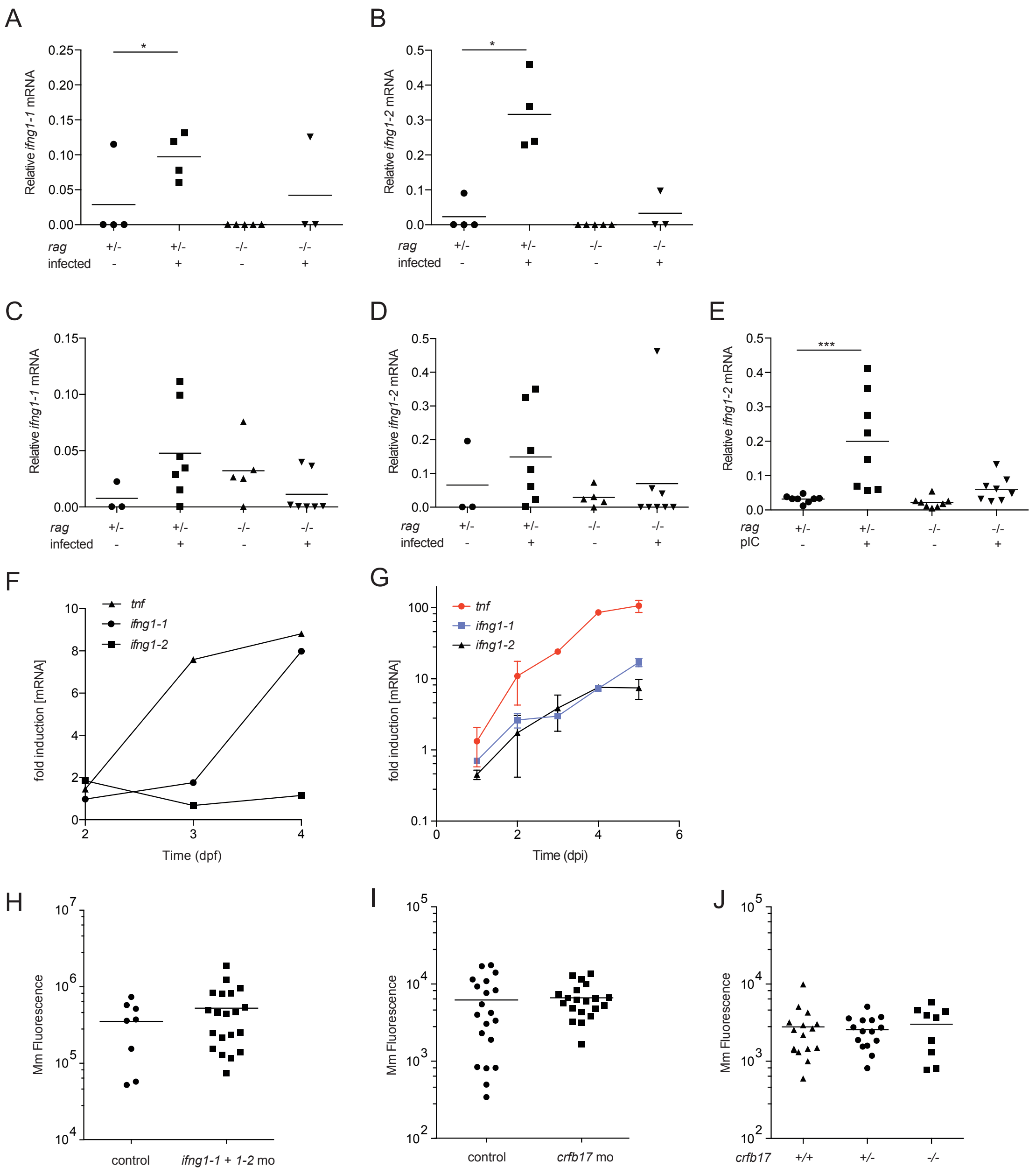

Figure S2. IFNγ induction does not occur in zebrafish larvae younger than 4dpf, related to Figure 2. (A, B) Real-time qPCR of *ifng1-1* (A) and *ifng1-2* (B) mRNA expression relative to *b-actin* in adult zebrafish at 6 weeks post-intraperitoneal infection with 20 CFU Mm in *rag* heterozygote and mutant adults. (C, D) Real-time qPCR of *ifng1-1* (C) and *ifng1-2* (D) mRNA expression relative to *b-actin* in adult zebrafish at 2 weeks post-intraperitoneal infection with 500 CFU Mm in *rag* heterozygote and mutant adults. (E) Real-time qPCR of *ifng1-2* mRNA expression relative to *b-actin* in adult zebrafish four hours after intraperitoneal injection with poly(I:C). (F) Real-time qPCR of *ifng1-1*, *ifng1-2* and *tnf* mRNA expression relative to *b-actin* in larval zebrafish four hours after intravenous injection with poly(I:C). (G)

Real-time qPCR of selected gene products following CV infection of 2dpf larvae with 275 CFU *M. marinum*. Shown for each point is an average of three experiments +/- SEM. (H) Bacterial burden measured at 4dpi following infection with 200 Mm in larvae treated at 0dpi with combined morpholinos targeting *ifng1-1* and *ifng1-2* or control. (I) Bacterial burden measured at 6dpi following infection with 200 Mm in larvae treated at 0dpi with a morpholino targeting *crfb17* or control. (J) Bacterial burden measured at 5dpi following infection with 250 Mm larvae from an incross of *crfb17* heterozygous (+/-) parents. Significance tested using ANOVA with Kruskal-Wallis multiple comparisons test. TNF induction was used as an independent indicator that adults and larvae were successfully infected. Each point in A-E represents one adult with the same adults tested in A-B and C-D. Each point in H-J represents one larva. Values in (F) and (G) represent pooled RNA from 30 larvae at each time point.

Figure S3

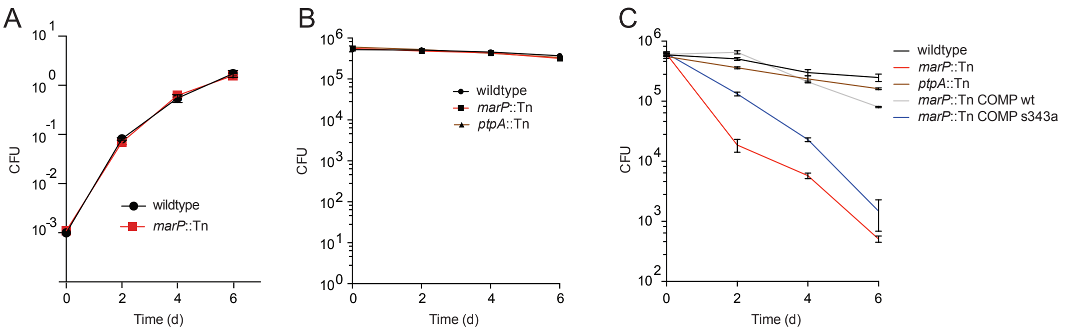

D

Strain	MIC (μg/mL)	
	Rif	Ery
wildtype	0.16	64
ΔESX-1	0.16	64
Δ*erp*	0.04	2
marP::Tn	0.02	2
ptpA::Tn	0.16	64

Figure S3. *marP*::Tn is unable to tolerate acidic environments and lipophilic antibiotics, related to Figure 3. (A) OD600 measurements of wild-type and *marP*::Tn in 7H9 media at neutral pH over 6 days of growth. (B) Wild-type, *marP*::Tn and *ptpA*::Tn bacteria were incubated for 6 days in phosphate-citrate buffer at pH 7 with CFU measurements taken every 2 days. (C) Wild-type, *marP*::Tn, *ptpA*::Tn, *marP*::Tn complemented with *marP* from Mtb and *marP*::Tn complemented with the *marP* S343A mutant were incubated in phosphate-citrate buffer at pH 4.5 for 6 days with CFU measurements taken every 2 days. (D) MIC measurements of hydrophobic antibiotics in wild-type, ΔESX-1, Δ*erp*, *marP*::Tn and *ptpA*::Tn. MICs were measured as described in Experimental Procedures and was determined as the minimum concentration that prevented the appearance of turbidity in culture. Rif, rifampin; Ery, erythromycin. Values in A, B, and C represent the mean +/- SD.

Figure S4

```
M. tuberculosis              PVTRDVYTIRADVEQGDSGGPLIDLNGQVLGVVFGAAID----DAETGFVLTA
M. bovis (99% identity)      PVTRDVYTIRADVEQGDSGGPLIDLNGQVLGVVFGAAVD----DAETGFVLTA
M. avium (86% identity)      PVTRDVYTIRASVEQGNSGGPLIDLNGQVLGVVFGAAVD----DPDTGFVLTA
M. elephantis (67% identity) TVEREVYTIRGTVRQGNSGGPMIDRDGNVLGVVFGAAVD----DADTGFVLTA
M. smegmatis (66% identity)  TVTREVYTVRGTVRQGNSGGPMINRAGKVLGVVFGAAVD----DVDTGFVLTA
M. marinum (86% identity)    TLNGLIQV-DAAIAPGDSGGPIVNNMGQVVGMNTAASDNFQMSGGGTGFAIPI
```

Figure S4. The serine protease encoded by MarP is highly conserved across mycobacterial species, related to Figure 4. Alignment of a section of MarP from Mtb (with active site serine S343 shown in red) with other species of mycobacteria. The overall identity between a given species and the entire gene from Mtb is reported in parentheses (alignments performed using LALIGN from NCBI database sequences).

Table S1. Prevalence of phagolysosomal trafficking of mycobacteria in vitro and in vivo

In vitro			
**Bacterium**	**Cell type**	**% phagosomes acidified (time)**	**Reference**
M. tuberculosis	Mouse peritoneal macrophages	36 (1 day)<br>23 (4 days)	Armstrong and Hart, 1971[7]
M. tuberculosis	Mouse peritoneal macrophages	23 (2 hours)	Hart *et al.,* 1972[8]
M. tuberculosis (immune rabbit serum treated)	Mouse peritoneal macrophages	79 (1 day)<br>68 (7 days)	Armstrong and Hart, 1975[9]
M. tuberculosis	Human primary mononuclear cells	25 (3 hours)<br>15 (22 hours)	Clemens and Horwitz 1995[10]
M. tuberculosis	Mouse bone marrow macrophages	10 (2 hours)	Pethe *et al*, 2004[11]
M. tuberculosis	Human macrophage cell line (THP-1)<br><br>THP-1 stimulated with IFNγ	10 (2 hours)<br><br>25 (2 hours)	Harris *et al,* 2008[12]
M. marinum	Mouse macrophage cell line (RAW)	21 (4 hours)<br>21 (8 hours)<br>27 (24 hours)	Barker *et al*, 1997[13]

In vivo			
**Bacterium**	**Host**	**% phagosomes acidified (time)**	**Reference**
M. tuberculosis	Mouse	30 (3 days)	Jayachandran *et al*., 2007[14]
M. tuberculosis	Human	30* (indeterminate)¢	Mwandumba *et al*., 2004[15]

Table S1. Related to Figures 1 and 2. Quantitation of lysosomal fusion of mycobacterial phagosomes as compiled from the literature. Note that in cases where bar graphs were shown for acidification instead of exact numbers, the extent was estimated to the nearest 5%.

*Estimated from Figure 6 of the paper

¢Patients presented with suspected pulmonary tuberculosis.

Supplemental Experimental Procedures

Bacterial Strains

Single-cell stocks were prepared as described for injection, and inocula were determined by injection onto selective 7H10 plates (Takaki et al., 2013). Heat-killed bacteria were prepared by incubating at 80°C for 20 minutes (Cambier et al., 2014). To make the transposon mutants, a transposon was created that contains an excisable hygromycin-resistance cassette to allow capture of transposon proximal sequences and this transposon was used to mutagenize wildtype Mm. The location of the transposon insertion in each gene was confirmed by sequencing prior to use. Wildtype Mm expressing EBFP2 under the *msp12* promoter was used as noted. All Mm strains were grown under hygromycin (Mediatech), streptomycin (Mediatech), or kanamycin (Mediatech) selection in Middlebrook's 7H9 medium (Difco) supplemented with glycerol, oleic acid, albumin, dextrose and Tween-80 (Takaki et al., 2013).

Mouse imaging

For ex vivo imaging studies, single cell lung preparations were made as previously described (Moguche et al., 2015). Infected (mCherry expressing) cells were isolated by FACS sorting with a FACSAria II (BD Biosciences, Franklin Lakes, NJ). Infected cells were then seeded onto 24-well glass-bottom plates coated with Cell-Tak adhesive (Corning) and incubated with 75nM LysoTracker Green DND-26 (Molecular Probes) for 30 minutes. Cells were washed and immediately imaged using an inverted Nikon microscope fitted with 20x and 40x objectives.

Zebrafish husbandry and injections

Larvae were maintained in fish water supplemented with *N*-phenylthiourea (PTU) from 1 day post-fertilization. The Tg(*mpeg1:YFP)*^{w200} line was used as previously described (Pagan et al., 2015). Larvae were randomly assigned to different experimental conditions, and were assigned after infection for all drug treatment studies. For the infectivity assays, larvae were infected in the HBV at 2.5dpf with 0.4 bacteria per injection. Heterozygous *rag1* mutant zebrafish were obtained from Artemis Pharmaceuticals (Köln, Germany) (Wienholds et al., 2002) and maintained as heterozygotes via outcrosses to the AB line. Carriers were incrossed to generate *rag1*-/- individuals. Genotype was determined at 3 to 6 months of age using DNA obtained from a tail clip procedure as described previously (Swaim et al., 2006) using a Taqman genotyping mix containing the following primers and probes:
rag1_Forward 5'-CTCAGAGTCAGCAGACGAACTG-3' , rag1_Reverse 5'-GGTTTCCATGAAAGGCTTAGCAAAA-3', rag1_WT Reporter 5'-CCTTTGACTCGGTCACG-3', rag1_ Mutant Reporter 5'-CCTTTGACTCAGTCACG-3'. Adult intraperitoneal injections were conducted as previously described (Cosma et al., 2006). Appropriate dilutions of single cell *M. marinum* stocks were used for infection so that 5μl would contain approximately 500 CFU (acute infection) or 20 CFU (chronic infection). Inoculum was confirmed with plate counts (Cosma et al., 2006). Crfb17 IFNγ receptor mutants (sa1747 Sanger Zebrafish Resource) was confirmed with sequencing and then High Resolution Melt Analysis using the same primers: crfb17_HRMF: 5'- TGTCTCGAGCAGCGTATAATG-3', crfb17_HRMR: 5'- GCTGCTTCCATGTTGATTGA – 3'. Intramacrophage burdens were enumerated as described (Takaki et al., 2013). Poly (I:C) - Polyinosinic-polycytidylic acid (poly I:C) (Sigma-Aldrich P1530) was dissolved in PBS buffer to a concentration of 5mg/mL and sterile filtered. For adult injections, 5μL of this solution vs 5μL PBS was injected intraperitoneally. For larval injections, 2dpf larvae were injected with approximately 10nL of poly (I:C) 5mg/mL solution in the caudal vein.

Microscopy

Fluorescence microscopy was performed as described (Pagan et al., 2015; Takaki et al., 2013). Quantification of bacterial burdens was performed using an inverted Nikon TiE microscope fitted with 4x and 10x objectives. For confocal microscopy, larvae were anesthetized in fish water with 0.025% Tricaine and embedded either in 1.5% low melting-point agarose or 2% hydroxymethylcellulose on optical bottom plates (MatTek). Confocal microscopy was done using a Nikon TiE microscope with 20x Plan Apo 0.75NA objective with A1 confocal system using a galvano scanner to generate 20-60μm stacks with 1-1.5μm vertical spacing. Photobleaching was performed using the 488nm laser on the confocal microscope with the minimal power and dwell time required to eliminate detection of bacterial fluorescence in the green

channel. Larvae were incubated at 28°C for 12 hours prior to assessing for fluorescence recovery. Data were acquired using NIS Elements version 4.4. Microscope scoring of staining, and bacterial enumeration, were performed in a blinded manner whenever possible.

Staining

LysoTracker Red DND-99 dye (DMSO solution) (Molecular Probes) was diluted 1:25 in PBS prior to injection of 5nL into the HBV or CV of larvae, which were then incubated for 1hr prior to imaging. MagicRed-Cathepsin (Immunochemistry Technologies) was resuspended at the concentration suggested by the manufacturer in DMSO, diluted 1:4 in 1xPBS prior to injection into the HBV or CV of larvae which were incubated for 1.5hr prior to imaging. pHrodo Green STP Ester and pHrodo Red succinimidyl ester (Molecular Probes) https://www.thermofisher.com/uk/en/home/references/molecular-probes-the-handbook/ph-indicators/probes-useful-at-acidic-ph.html#head4 were resuspended in DMSO according to manufacturer instructions and stored in the dark at -20°C until use. Thawed single cell preparations of *M. marinum* were resuspended in 200µL 1xPBS and incubated with 1µL pHrodo Green or Red for 30 min at 30°C. The bacteria were washed once with 1xPBS and resuspended in PBS pH 7.4 prior to injection into the zebrafish, or resuspended in phosphate-citrate buffer of appropriate pH for in vitro studies. For single-cell infections, the bacteria were confirmed to be pHrodo-negative at the time of dose confirmation.

Morpholinos

Morpholino oligonucleotides (mo) were designed and synthesized by Gene Tools (Philomath, OR). Morpholinos were diluted in 1x Buffer Tango (ThermoFisher) 2% phenol red sodium salt solution (Sigma) and injected 1nL into the yolk of 1 cell-stage embryos. *atp6v1a* translation blocking morpholino sequence: ATCCATCTTGTGTGTTAGAAAACTG as described (Horng et al., 2007). *tnfr1* morpholino was used as described (Roca and Ramakrishnan, 2013). *tnfr1* morpholino targeting the exon 5 / intron 6 boundary: CTGCATTGTGACTTACTTATCGCAC. *crfb17* splice blocking morpholino (GQ901865) TTAAACTAAATCGCCTTACCTTGTG, *ifng1* (NM_001020793) AAAAGAATACTGACCAGCATAGATG and *ifng2* (NM_212864) TGAAGGCGTTCGCTAAAGTTAGAGT were used as described (Aggad et al., 2010).

Drug treatments

A stock of Bafilomycin A1 (Cambridge Bioscience) was dissolved in DMSO prior to use. Larvae were treated with Baf at 50nM in 0.5% DMSO via soaking at 2dpf following infection and were assessed at 24-40 hpi.

RNA isolation and quantitative real-time PCR (qRT-PCR)

Adult fish were euthanized according to approved Animal Use Protocols. Fish were then flash-frozen with liquid nitrogen and homogenized using a mortar and pestle in 5ml Trizol reagent (Life Technologies). A 1mL aliquot was then further processed according to the manufacturer's protocol. Larval zebrafish were euthanized according to approved Animal Use Protocols. Total RNA from batches of ~30 embryos per biological replicate was isolated with TRIzol Reagent (Life Technologies) and used to synthesize cDNA with Superscript II reverse transcriptase and oligo-dT primers (Invitrogen). Quantitative RT-PCR was performed as previously described (Clay et al., 2007) with SYBR green PCR Master Mix (Applied Biosystems) on an ABI Prism 7300 Real Time PCR System (Applied Biosystems). Each biological replicate was run in triplicate, and average values were plotted. Data were normalized to *b-actin* for $\Delta\Delta$Ct analysis. The primers used in this study were: ifng1-1 Forward = 5'-ATTCCTGCCTCAAAATGGTG-3', ifng1-1 Reverse 5' – TTTTCCAACCCAATCCTTTG-3', ifng1-2 Forward 5'- CTATGGGCGATCAAGGAAAA-3', ifng1-2 Reverse 5'= CTTTAGCCTGCCGTCTCTTG-3', bactin Forward 5'-ACCTCATGAAGATCCTGACC-3', bactin Reverse 5'-TGCTAATCCACATCTGCTGG-3', tnf Forward 5'-AGGCAATTTCACTTCCAAGG-3', tnf Reverse 5'-CAAGCCACCTGAAGAAAAGG-3'

MIC assays

MICs for each strain were determined in 7H9 broth. A suspension of single bacteria was inoculated into 7H9 ($2x10^4$ ml^{-1}) containing antibiotics at different concentration and cultured for 6 days before scoring for

turbidity with each concentration tested in triplicate. The MIC was defined as the minimal concentration at which turbidity was not observed.

Supplemental References

Aggad, D., Stein, C., Sieger, D., Mazel, M., Boudinot, P., Herbomel, P., Levraud, J.-P., Lutfalla, G., and Leptin, M. (2010). In Vivo Analysis of Ifn-g1 and Ifn-g2 Signalling in Zebrafish. Journal of Immunology *185*.

Cambier, C.J., Takaki, K.K., Larson, R.P., Hernandez, R.E., Tobin, D.M., Urdahl, K.B., Cosma, C.L., and Ramakrishnan, L. (2014). Mycobacteria manipulate macrophage recruitment through coordinated use of membrane lipids. Nature *505*, 218-222.

Clay, H., Davis, J.M., Beery, D., Huttenlocher, A., Lyons, S.E., and Ramakrishnan, L. (2007). Dichotomous role of the macrophage in early Mycobacterium marinum infection of the zebrafish. Cell Host and Microbe *2*, 29-39.

Cosma, C.L., Swaim, L.E., Volkman, H., Ramakrishnan, L., and Davis, J.M. (2006). Zebrafish and frog models of Mycobacterium marinum infection. Curr Protoc Microbiol *Chapter 10*, Unit 10B.12.

Horng, J.L., Lin, L.Y., Huang, C.J., Katoh, F., Kaneko, T., and Hwang, P.P. (2007). Knockdown of V-ATPase subunit A (atp6v1a) impairs acid secretion and ion balance in zebrafish (Danio rerio). Am J Physiol Regul Integr Comp Physiol *292*, R2068-2076.

Moguche, A.O., Shafiani, S., Clemons, C., Larson, R.P., Dinh, C., Higdon, L.E., Cambier, C.J., Sissons, J.R., Gallegos, A.M., Fink, P.J*., et al*. (2015). ICOS and Bcl6-dependent pathways maintain a CD4 T cell population with memory-like properties during tuberculosis. The Journal of experimental medicine *212*, 715-728.

Pagan, A.J., Yang, C.T., Cameron, J., Swaim, L.E., Ellett, F., Lieschke, G.J., and Ramakrishnan, L. (2015). Myeloid Growth Factors Promote Resistance to Mycobacterial Infection by Curtailing Granuloma Necrosis through Macrophage Replenishment. Cell Host Microbe *18*, 15-26.

Roca, F.J., and Ramakrishnan, L. (2013). TNF dually mediates resistance and susceptibility to mycobacteria via mitochondrial reactive oxygen species. Cell *153*, 521-534.

Swaim, L.E., Connolly, L.E., Volkman, H.E., Humbert, O., Born, D.E., and Ramakrishnan, L. (2006). Mycobacterium marinum infection of adult zebrafish produces caseating granulomatous tuberculosis and is moderated by adaptive immunity. Infect Immun *74*, 6108-6117.

Takaki, K., Davis, J.M., Winglee, K., and Ramakrishnan, L. (2013). Evaluation of the pathogenesis and treatment of Mycobacterium marinum infection in zebrafish. Nat Protoc *8*, 1114-1124.

Wienholds, E., Schulte-Merker, S., Walderich, B., and Plasterk, R.H. (2002). Target-selected inactivation of the zebrafish rag1 gene. Science *297*, 99-102.
