# Supplementary material for: Mycobacterial Acid Tolerance Enables Phagolysosomal Survival and Establishment of Tuberculous Infection In Vivo
Source: Cell Host Microbe. 2016 Aug 10;20(2):250–8. doi: 10.1016/j.chom.2016.07.007 (PMC4985559; doi:10.1016/j.chom.2016.07.007)
Supplement: Document S2. Article plus Supplemental Information [file mmc2.pdf]

Short Article

Cell Host & Microbe

Mycobacterial Acid Tolerance Enables Phagolysosomal Survival and Establishment of Tuberculous Infection In Vivo

Graphical Abstract

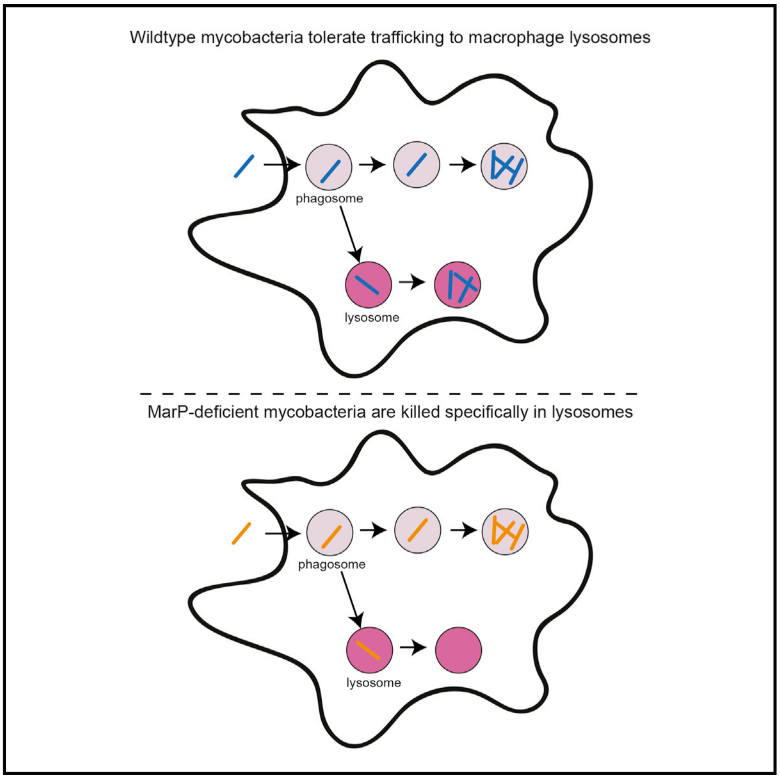

Authors

Steven Levitte, Kristin N. Adams, Russell D. Berg, Christine L. Cosma, Kevin B. Urdahl, Lalita Ramakrishnan

Correspondence

lr404@cam.ac.uk

In Brief

Blocking phagolysosomal fusion is considered critical for mycobacterial survival within macrophages. Levitte et al. (2016) show that while a substantial proportion of macrophage infecting mycobacteria are trafficked to lysosomes in vivo, the acid tolerance determinant MarP enables them to survive and replicate within lysosomes, thereby enhancing their ability to establish infection.

Highlights

- In vivo, newly infecting mycobacteria are rapidly trafficked to lysosomes within macrophages
- The mycobacterial acid tolerance determinant MarP enables lysosomal survival and growth
- Phagolysosomal mycobacteria can successfully establish infection, which is MarP dependent

Levitte et al., 2016, Cell Host & Microbe *20*, 250–258
August 10, 2016 © 2016 The Authors. Published by Elsevier Inc.
http://dx.doi.org/10.1016/j.chom.2016.07.007

Cell Host & Microbe
Short Article

Mycobacterial Acid Tolerance Enables Phagolysosomal Survival and Establishment of Tuberculous Infection In Vivo

Steven Levitte,[1,2] Kristin N. Adams,[3,4] Russell D. Berg,[2] Christine L. Cosma,[5] Kevin B. Urdahl,[3,6] and Lalita Ramakrishnan[1,*]
[1]Molecular Immunity Unit, Department of Medicine, University of Cambridge, MRC Laboratory of Molecular Biology, Cambridge CB2 0QH, UK
[2]Molecular and Cellular Biology Graduate Program and Medical Scientist Training Program, University of Washington, Seattle, USA
[3]Center for Infectious Disease Research, Seattle, WA 98195, USA
[4]Department of Pediatrics
[5]Department of Microbiology
[6]Department of Immunology
University of Washington, Seattle, WA 98109, USA
*Correspondence: lr404@cam.ac.uk
http://dx.doi.org/10.1016/j.chom.2016.07.007

SUMMARY

The blockade of phagolysosomal fusion is considered a critical mycobacterial strategy to survive in macrophages. However, viable mycobacteria have been observed in phagolysosomes during infection of cultured macrophages, and mycobacteria have the virulence determinant MarP, which confers acid resistance in vitro. Here we show in mice and zebrafish that innate macrophages overcome mycobacterial lysosomal avoidance strategies to rapidly deliver a substantial proportion of infecting bacteria to phagolysosomes. Exploiting the optical transparency of the zebrafish, we tracked the fates of individual mycobacteria delivered to phagosomes versus phagolysosomes and discovered that bacteria survive and grow in phagolysosomes, though growth is slower. MarP is required specifically for phagolysosomal survival, making it an important determinant for the establishment of mycobacterial infection in their hosts. Our work suggests that if pathogenic mycobacteria fail to prevent lysosomal trafficking, they tolerate the resulting acidic environment of the phagolysosome to establish infection.

INTRODUCTION

As first-line immune defense cells, macrophages phagocytose invading microbes, delivering them to lysosomes for degradation (Huynh and Grinstein, 2007). Therefore, to survive intracellularly, pathogens must avoid phagosomal fusion with lysosomes, survive within lysosomal compartments, or escape out of the phagosome to reside in the cytosol (Asrat et al., 2014). Studies in cultured macrophages have found that mycobacteria are capable of occupying all these subcellular niches (Cambier et al., 2014a).

Active avoidance of phagosome-lysosome fusion has been noted as a significant mycobacterial survival strategy, particularly during the innate immune phase of infection (Asrat et al., 2014; Behar and Baehrecke, 2015; Kasper et al., 2015; MacMicking, 2008; Tan and Russell, 2015). The idea that phagosomal blockade is integral to *Mycobacterium*'s intracellular survival and growth has been bolstered by findings that pretreatment of macrophages with gamma-interferon (IFNγ), a cytokine produced predominantly during the adaptive immune response, increases phagolysosomal fusion and decreases bacterial survival (Schaible et al., 1998). These findings led to the conclusion that IFNγ increases macrophage microbicidal capacity by enhancing mycobacterial trafficking to lysosomes (see Table S1 available online) (Flynn and Chan, 2001; Schaible et al., 1998; Via et al., 1998). However, IFNγ enhances macrophage killing through multiple mechanisms (Nunes-Alves et al., 2014), and dead mycobacteria are trafficked to macrophage lysosomes independently of IFNγ (Armstrong and Hart, 1975; Barker et al., 1997). Therefore, the enhanced killing mediated by IFNγ might be the cause of increased lysosomal trafficking rather than the effect.

Furthermore, three lines of evidence suggest that mycobacteria can tolerate lysosomal trafficking: (1) multiple studies find a substantial proportion of infecting *M. tuberculosis* (Mtb) in phagolysosomes soon after infection of cultured macrophages, with 10%–25% at 2–3 hr and 15%–36% at 24 hr (Table S1); (2) Mtb survives and even replicates upon delivery into lysosomes, either through Fc receptor-mediated phagocytosis or by co-infection with the lysosomal pathogen *Coxiella burnetti* (Armstrong and Hart, 1975; Gomes et al., 1999); and (3) the membrane serine protease Rv3671c (MarP), which was identified in an in vitro screen for acid tolerance determinants, is also required for virulence (Small et al., 2013; Vandal et al., 2008), consistent with the idea that Mtb experiences acid stress in vivo.

The macrophage lysosomal avoidance and tolerance strategies employed by mycobacteria have been described for cultured macrophages (Table S1). Here, we directly address in vivo the prevalence and consequences of lysosomal trafficking using the optically transparent zebrafish larva, which allows for real-time tracking of infection with *M. marinum* (Mm), a

This is an open access article under the CC BY license (http://creativecommons.org/licenses/by/4.0/).

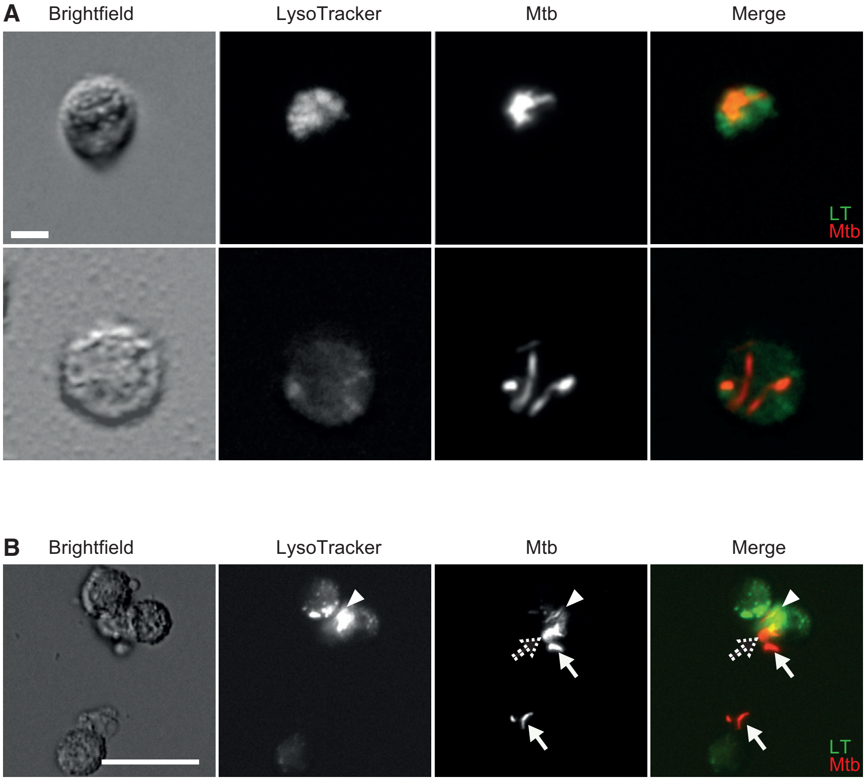

Figure 1. Direct Ex Vivo Phagolysosomal Localization of *M. tuberculosis*

Images of red fluorescent Mtb H37Rv from infected lung. Cells were sorted from lung tissue at 13 days post-infection (dpi) (A) or 19 dpi (B) and stained with LysoTracker (LT) Green dye.

(A) The top images show an infected macrophage in which the Mtb colocalizes with LT, while the bottom images show an infected macrophage in which the Mtb does not colocalize with LT. Representative of two mice. Scale bar, 5 μm.

(B) Arrowheads depict bacteria that colocalize with LT, while arrows depict bacteria that do not colocalize with dye. The dotted arrow marks partially colocalized bacteria. Representative of three mice. Scale bar, 25 μm.

close genetic relative of Mtb and a natural agent of tuberculosis (TB) in ectotherms (Cambier et al., 2014a). We find that innate macrophages, in the absence of IFNγ stimulation, deliver a substantial proportion of infecting mycobacteria to lysosomes. However, this potentially host-beneficial innate immune strategy is effectively counteracted by mycobacterial MarP, which specifically mediates bacterial survival and growth within lysosomes.

RESULTS

Mtb Resides in Macrophage Phagosomes and Phagolysosomes after Aerosol Infection of Mice

Macrophages derived from a variety of hosts rapidly traffic a proportion of infecting mycobacteria to lysosomes in vitro (Table S1). We asked whether this was also the case in vivo during the first macrophage-mycobacterium interaction, which in humans and mice is thought to occur within lung-resident macrophages (Verrall et al., 2014). We infected mice with ~200 fluorescent Mtb by aerosolization and assessed lysosomal trafficking as judged by bacterial co-localization with LysoTracker, an acidophilic dye that labels lysosomes. We chose 13 and 19 days post-infection, time points that flank the 14–15 day time point when IFNγ-producing T cells begin to arrive in the lung after aerosol Mtb infection (Khader et al., 2007). (Analysis before 13 days was precluded by the rarity of Mtb-infected cells.) Mtb in lysosomal compartments was readily observed in all animals (two at 13 days and three at 19 days) (Figure 1), though the relative rarity of infected cells even at these time points precluded quantification of the extent of lysosomal trafficking. Because lung-resident macrophages are the predominant infected cell type early after aerosolized Mtb infection (Urdahl, 2014; Wolf et al., 2007), our findings suggest that these first-responding cells can traffic Mtb to lysosomes in the sole context of innate immunity. A human study of HIV-positive TB patients also found ~30% of Mtb in phagolysosomes of alveolar macrophages obtained by bronchoalveolar lavage, although the duration of infection was unknown in this case (Table S1) (Mwandumba et al., 2004).

A Proportion of Mm Is Rapidly Trafficked to Lysosomes after Infection of Larval Zebrafish

To probe lysosomal trafficking of mycobacteria in vivo and its consequences in real time, we turned to zebrafish larvae infected with Mm. In cultured macrophages, a similar proportion of Mm (21% at 4 hr post-infection [hpi]) as Mtb is rapidly trafficked to lysosomes (Table S1) (Barker et al., 1997). We infected zebrafish larvae with fluorescent Mm in the hindbrain ventricle (HBV), an epithelium-lined cavity to which myeloid cells are rapidly recruited in response to infecting Mm (Cambier et al., 2014b) (Figure 2A). Using LysoTracker, which has been shown to label lysosomes in larval zebrafish (Peri and Nüsslein-Volhard, 2008), we found that a substantial proportion of the bacteria was in acidified compartments within 24 hpi (Figures 2B and 2C). Similar results were obtained after injecting bacteria into the caudal vein (CV), which traverses the hematopoietic tissue, the site of intermediate myelopoiesis giving rise to circulating monocytes (Clements and Traver, 2013) (Figure 2A); mycobacteria were rapidly and progressively delivered to acidified compartments of circulating myeloid cells within 3 to 24 hpi (Figure 2D). In vitro, the mycobacterial phagosome has been reported to be only slightly acidified to a pH of 6.2 (Tan and Russell, 2015), so we asked whether the bacteria colocalizing with LysoTracker were in slightly acidified phagosomes or in bona fide lysosomal compartments, which have a pH <5 and contain hydrolytic enzymes. We directly assessed the pH at the bacterial surface by labeling Mm prior to infection with pHrodo, a pH-sensitive dye. Incubation of pHrodo-stained Mm in phosphate-citrate buffer

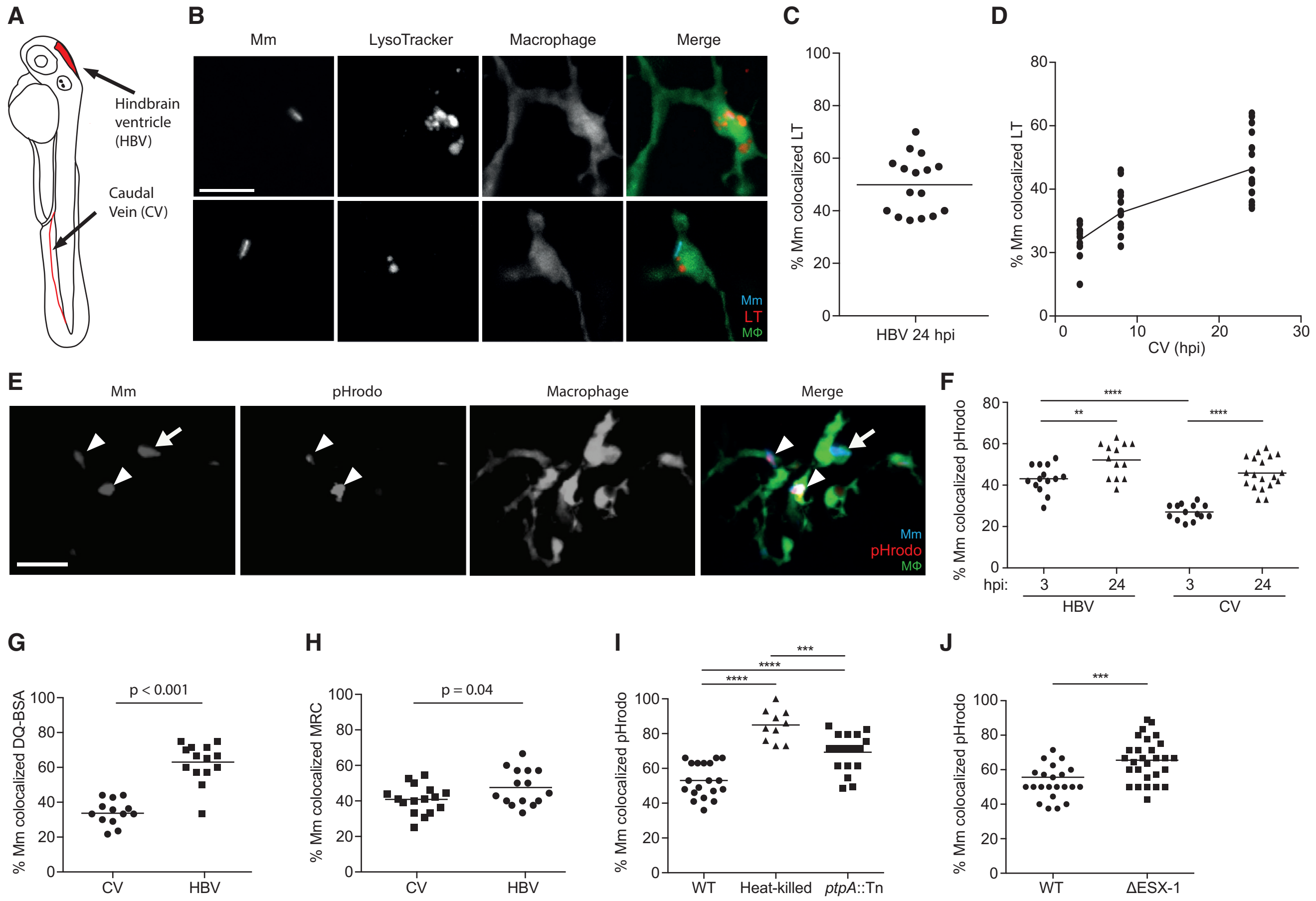

Figure 2. In Vivo Phagolysosomal Trafficking of *M. marinum*

(A) Illustration of zebrafish larva with injection sites outlined in red. The hindbrain ventricle (HBV) is accessible to recruited myeloid cells, while the caudal vein (CV) traverses the caudal hematopoietic tissue where myeloid cells develop.

(B) Confocal images of blue fluorescent Mm that have been phagocytosed by green fluorescent macrophages in the brain of a 2 day post-fertilization (dpf) larva stained with red LT; one bacterium shown colocalizes with LT, and the other does not. Scale bar, 10 μm.

(C) Percent of Mm colocalizing with LT dye 24 hr post infection (hpf) into the HBV, representative of three experiments.

(D) Percent of Mm colocalizing with LT dye at 3, 8, and 24 hpi in the CV, representative of three experiments.

(E) Confocal images of blue fluorescent Mm that were pre-labeled with red pHrodo prior to infection into the HBV of 2dpf larvae with green fluorescent macrophages; bacteria that colocalize with pHrodo (arrowheads) and one that does not (arrow). Scale bar, 10 μm.

(F) Percent of Mm colocalizing with pHrodo at 3 and 24 hpi in the HBV or CV of a 3 dpf larva, representative of two experiments.

(G and H) Percent of Mm colocalizing with DQ-BSA (G) or MR-Cathepsin (MRC) (H) imaged at 24 hpi following infection in the HBV or CV of 2 dpf larvae, representative of two experiments each.

(I) Percent of live, heat-killed, or ptpA::Tn Mm pre-labeled with pHrodo prior to infection into the HBV of 2 dpf larvae imaged at 24 hpi, representative of three experiments.

(J) Percent of ΔESX-1 Mm colocalizing with pHrodo imaged at 24 hpi in the HBV of 2 dpf larvae, representative of three experiments. Significance tested using one-way ANOVA with Tukey's post-test (F and I) or two-tailed unpaired t test (G, H, and J). Each point in (C), (D), and (F)–(J) represents one larva, with mean depicted as a horizontal line. See also Figures S1 and S2.

at a range of pH values showed that fluorescence was greatly increased in bacteria experiencing a pH of ≤ 5.0 (Figure S1A). We used a fluorescence intensity cutoff for imaging pHrodo-stained bacteria in the zebrafish so as to exclude those experiencing pH >5.0 (Figure S1A). pHrodo-stained bacteria were detected using this cutoff soon after both HBV and CV infection (Figures 2E and 2F), and co-staining with LysoTracker and pHrodo showed strong overlap (Figures S1B and S1C). Staining with MR-Cathepsin and DQ-BSA, fluorogenic protease substrates that fluoresce only upon hydrolysis and label macrophage lysosomes in zebrafish (Peri and Nüsslein-Volhard, 2008), revealed a similar proportion of phagolysosome-localized bacteria as pHrodo staining (Figures 2G and 2H). Thus, during in vivo infection, macrophages rapidly deliver a proportion of mycobacteria to bona fide lysosomal compartments characterized by hydrolase activity and pH <5.

In the context of cultured macrophages, the observed distribution of Mtb and Mm in phagosomes versus phagolysosomes is the result of counteracting macrophage and bacterial strategies. While the macrophage can consign a significant proportion to the lysosome, the majority of bacteria actively avoid this fate, as evidenced by the finding that killed bacteria are rapidly

trafficked to lysosomes (Armstrong and Hart, 1971; Barker et al., 1997). Multiple determinants have been implicated in Mtb's avoidance of lysosomes, e.g., the tyrosine phosphatase PtpA (Bach et al., 2008), and the specialized secretion system ESX-1 (MacGurn and Cox, 2007). To further validate our findings in zebrafish macrophages, we first asked if Mm actively avoids lysosomal trafficking by comparing pHrodo-stained live and heat-killed bacteria (Figure 2I). We observed increased lysosomal localization of killed Mm. Moreover, both a Mm *ptpA* (*mmar_3309*) transposon insertion mutant and an ESX-1-deficient mutant displayed increased lysosomal localization albeit less than heat-killed bacteria (Figures 2I and 2J), suggesting the presence of additional mycobacterial factors that mediate phagolysosomal blockade, as has been reported for Mtb (Asrat et al., 2014).

In sum, our findings suggest that Mm actively blocks phagosome-lysosome fusion during zebrafish infection through mechanisms similar to those used by Mtb in cultured macrophages. But similarly to observations in cultured macrophages, this blockade is only partially successful. Conversely, macrophages, regardless of species, are robustly equipped to consign a substantial proportion of infecting mycobacteria to lysosomal compartments.

Trafficking of Mycobacteria to Lysosomes Does Not Require IFNγ

Our observed proportions of phagolysosomal bacteria in vivo at 24 hpi (45%–52%) were a little higher than the 15%–36% reported for Mtb and Mm in cultured macrophages (Figures 2C and 2D; Table S1). Since IFNγ stimulates phagosome-lysosome fusion in cultured macrophages (Schaible et al., 1998; Via et al., 1998), we asked if it was responsible for the small increase in lysosomal trafficking observed in vivo. In mammals, IFNγ is predominantly produced by T lymphocytes, which have not yet developed in the zebrafish larvae (Clements and Traver, 2013); however, there are also innate sources of IFNγ (e.g., natural killer cells) (Renshaw and Trede, 2012). Both zebrafish IFNγ orthologs—*ifng1-1* and *ifng1-2*—were induced largely in a RAG-dependent fashion at 6 weeks post-infection in adult zebrafish. This result is similar to mouse Mtb infection, where adaptive immune cells are the predominant source of IFNγ (Baldridge et al., 2010; Flynn and Chan, 2001) (Figures S2A and S2B). Similar trends were observed by 2 weeks post-infection in adults (Figures S2C and S2D). To look for innate sources of IFNγ with a more sensitive assay, we tested *ifng1-2* expression in response to a potent IFNγ inducer, the TLR3 agonist poly(I:C) (Aggad et al., 2010) (Figure S2E). We confirmed its rapid induction (4 hr post-administration) in adult animals from both innate and adaptive immune sources, but predominantly the latter (Figure S2E). In the larva also, we observed poly(I:C)-mediated IFNγ induction starting at 4 days postfertilization but failed to detect induction of either homolog earlier in development (Figure S2F). This pattern held up for Mm infection: neither *ifng1-1* nor *ifng1-2* induction was detected before 4 days post-fertilization (2 days post-infection) (Figure S2G). In sum, our data suggest that zebrafish induce IFNγ in response to Mm similarly to mice responding to Mtb. While some IFNγ can be made by innate sources, these innate cells have yet to mature during the window used in our studies on phagolysosomal fusion. While we failed to observe induction of IFNγ at early time points, this does not exclude small amounts being present from early during development. We addressed the potential contribution of a small amount of IFNγ by assessing infection in larvae injected with *ifng1-1* and *ifng1-2* morpholinos, *crfb17* morpholino (part of the signaling machinery for both IFNγ1 and IFNγ2), and a mutant in *crfb17* (Aggad et al., 2010; Sieger et al., 2009). We did not observe hypersusceptibility to infection in any of these conditions (Figures S2H–S2J). In sum, this early phagolysosomal fusion likely represents the intrinsic ability of the macrophage to deliver infecting bacteria to lysosomal compartments.

The Host-Protective Effect of Lysosomal Trafficking Is Limited through the Action of the Mycobacterial Serine Protease MarP

Studies in cultured macrophages have come to different conclusions about the consequences of mycobacterial phagolysosomal trafficking depending on the stimulus used to achieve it. Mycobacteria delivered into phagolysosomes by opsonization or by co-infection with the lysosomally localized bacterium *Coxiella burnetti* survived and even replicated in lysosomes, whereas bacteria reaching lysosomes following IFNγ stimulation of the macrophages were killed (Armstrong and Hart, 1975; Cosma et al., 2003; Gomes et al., 1999). To test macrophage-intrinsic phagosomal maturation as a microbicidal effector mechanism in vivo, we disrupted phagosomal maturation by treating larvae with the vATPase inhibitor Bafilomycin (Peri and Nüsslein-Volhard, 2008), or used a translation-blocking morpholino targeting *atp6v1a*, a subunit required for vATPase function in the zebrafish (Horng et al., 2007). LysoTracker and pHrodo staining each confirmed disruption of lysosomal localization in the context of infection (Figures 3A–3C). Bafilomycin treatment and *atp6v1a* knockdown each increased bacterial burdens within 24 hr (Figures 3D and 3E). This increase in bacterial burdens was reflected in increased bacterial replication within individual larval macrophages (Figure 3F), similar to the reduced microbicidal capacity of TNF-deficient macrophages (Figure 3F) (Clay et al., 2008). Thus, phagosomal maturation restricts the progression of infection by decreasing intramacrophage replication of Mm.

The fact that Mm infection progresses overall despite growth restriction mediated by the lysosome suggested one of two explanations: either that the expansion of overall infection is driven exclusively by the bacterial population avoiding lysosomes, or that bacteria can replicate in both phagosomes and lysosomes. To distinguish between these possibilities, we used two mutants—a transposon mutant in the ortholog (*mmar_5159*) of the Mtb membrane serine protease MarP, which is required for acid tolerance in vitro and virulence in mice (Vandal et al., 2008), and *ptpA*::Tn, which displays increased lysosomal trafficking (Figure 2I). We confirmed that *marP*::Tn was acid sensitive and hypersusceptible to lipophilic antibiotics in vitro, and attenuated in vivo (Figures S3A–S3D and Figure 3G), consistent with the phenotypes of its Mtb counterpart (Vandal et al., 2008). Because of MarP's multiple in vitro phenotypes (increased susceptibility to acid, hydrophobic antibiotics and detergents, reactive oxygen species, and nitric oxide), it has not been clear which of these is responsible for its attenuation in vivo (Ehrt et al., 2015; Stallings and Glickman, 2010). We reasoned that if defective lysosomal tolerance contributes to *marP*::Tn attenuation, then

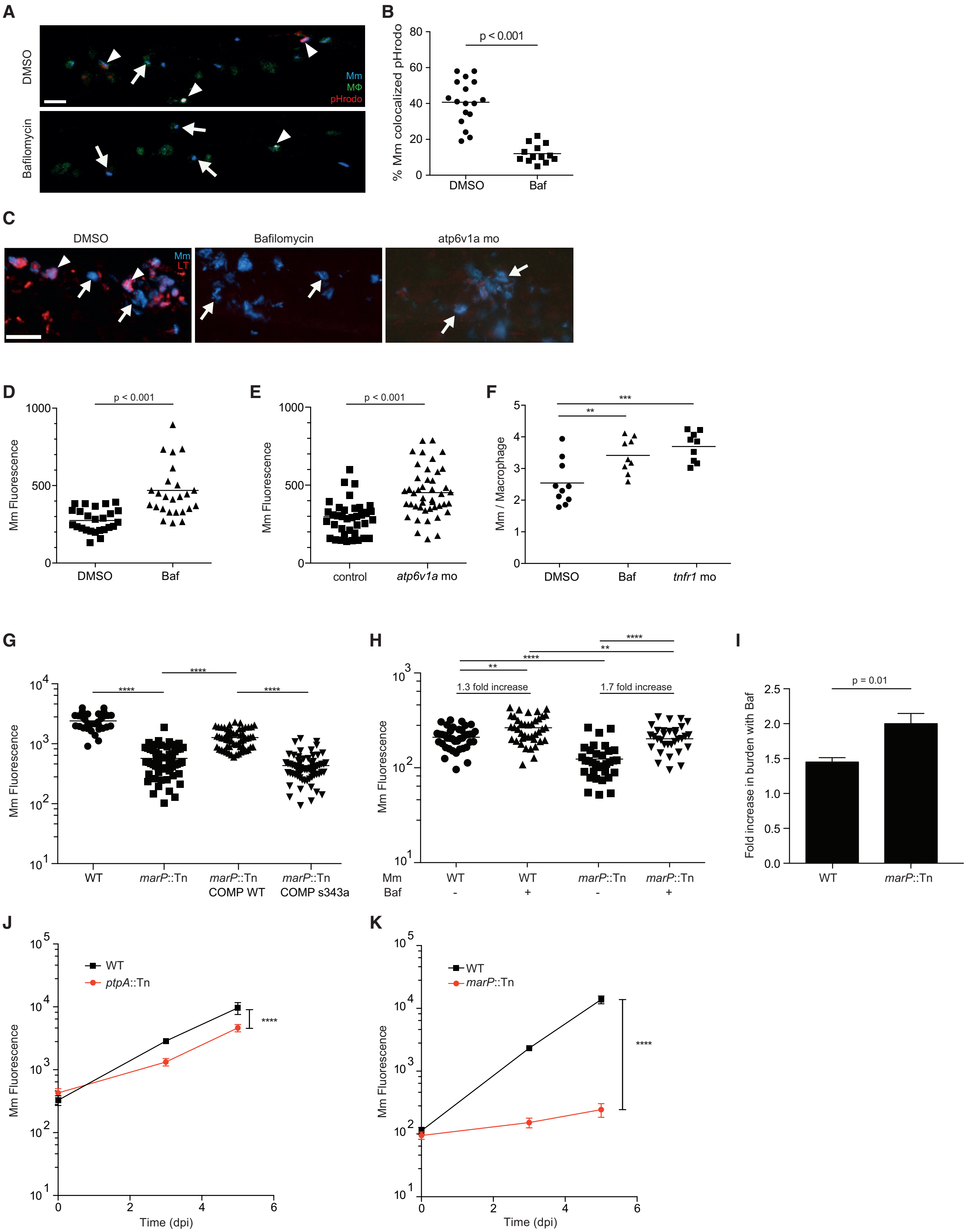

(legend on next page)

Bafilomycin should enhance *marP*::Tn growth more than wild-type. It did (2.0- ± 0.15-fold increase in burden for *marP*::Tn versus 1.5- ± 0.06-fold for wild-type, $p = 0.01$) (Figures 3H and 3I). Furthermore, *marP*::Tn was ~25 times more attenuated than *ptpA*::Tn during a 5-day infection (Figures 3J and 3K), suggesting that, in vivo, tolerance of the acidic lysosomal environment is a more significant determinant of mycobacterial growth than avoidance of phagosome-lysosome fusion. This is consistent with findings in the mouse-Mtb aerosol infection model where the MarP mutant is severely attenuated but the PtpA mutant is not (Grundner et al., 2008; Vandal et al., 2008). In sum, lysosomal trafficking is a host-beneficial strategy that limits intramacrophage mycobacterial growth and thereby expansion of infection in the granuloma. The effectiveness of this strategy is substantially offset, however, by MarP-mediated tolerance of lysosomal trafficking.

Mycobacteria Delivered to Phagolysosomes Can Successfully Establish Infection in a MarP-Dependent Fashion

We probed the basis of lysosomal trafficking as a host-protective mechanism (bactericidal, bacteriostatic, or simply slowing bacterial growth) and, conversely, how mycobacterial MarP counters this strategy. The optical transparency of the zebrafish, particularly its HBV, allowed us to map the fate of individual mycobacteria following initial distribution into phagosomes versus phagolysosomes. We injected single pHrodo-labeled bacteria into the HBV (Figure 4A), sorted the animals based on whether the bacteria were found within pHrodo-labeled phagolysosomes at 12 hpi, and then tracked bacterial fates for 48 hr to determine if the host was still infected based on the presence of fluorescent Mm in the HBV (Figure 4A). The fraction of infected larvae at 60 hpi was not significantly different between the phagosomal and phagolysosomal groups (Figure 4B). We confirmed that the bacteria were alive and metabolically active by photobleaching them and assessing for recovery of fluorescence (Figures 4C and 4D). In infected animals, we assessed the extent of bacterial growth in phagosomes versus phagolysosomes by enumerating the number of bacteria at 60 hpi. Mm replicated within the phagolysosome, though at a slower rate than in the non-acidified phagosome (Figure 4E).

Consistent with a primary role of MarP in tolerating the lysosomal environment, *marP*::Tn was cleared more than wild-type only when it was trafficked to the phagolysosome (Figure 4F). Individual mutant bacteria that were in non-acidified phagosomes survived similarly to wild-type (compare Figures 4F and 4D). In contrast, *ptpA*::Tn and ΔESX-1, which are preferentially trafficked to the phagolysosome but are not sensitive to acid in vitro, survived in either compartment similarly to wild-type (Figures 4G and 4H).

In sum, these experiments suggest that lysosomal residence is fully conducive to mycobacterial survival and also supports growth, albeit at a slower rate than nonacidified phagosomes. Mycobacterial MarP mediates this lysosomal survival.

DISCUSSION

Our in vivo work detailing the progression of mycobacterial trafficking in macrophages and its consequences suggests that pathogenic mycobacteria encounter and counter macrophage phagolysosomal fusion from the earliest stage of infection. The lysosomal avoidance and tolerance strategies utilized by mycobacteria are not mutually exclusive, and our findings suggest that newly arriving mycobacteria employ a tiered strategy to successfully establish infection in first-responder macrophages. In the face of frequently successful phagosome-lysosome fusion by these macrophages, mycobacteria must fall back on their ability to contend with the microbicidal arsenal of the lysosome (Vandal et al., 2009). The increased lysosomal localization (40%–50%) we observe in vivo over cultured macrophages (15%–36%) may reflect the enhancement of intrinsic macrophage phagolysosomal fusion capacity by cues in vivo which must derive from innate immunity, since they are independent of both IFNγ and Fc receptor (i.e., antibodies).

In terms of the consequences of phagolysosomal fusion, we find that it does not enhance the macrophage's ability to kill

Figure 3. Lysosomal Trafficking Is a Host-Beneficial Process, which Is Counteracted by Bacterial MarP

(A) Confocal images of 3 dpf larvae that were infected with Mm in the CV at 2 dpf and then treated for 24 hr with 50 nM Bafilomycin (Baf) or DMSO control. Arrowheads denote bacteria labeled with pHrodo, while arrows show pHrodo-negative bacteria.

(B) Percent of Mm colocalizing with pHrodo at 24 hpi as shown in (A), representative of two experiments.

(C) Confocal images of 3 dpf larvae that were infected in the CV with blue fluorescent Mm at 2 dpf and stained with red LT at 3 dpf following treatment with DMSO, 50 nM Baf, or morpholino targeting *atp6v1a* at 0 dpf. Arrowheads denote bacteria that colocalize with LT, while arrows denote LT-negative bacteria.

(D) Bacterial burden (FPC) measured at 24 hpi with 300 Mm; fish were randomly assigned to DMSO or 50 nM Baf treatment immediately after infection, representative of three experiments.

(E) Bacterial burden measured at 24 hpi with 300 Mm in larvae injected at 0 dpf with a morpholino targeting *atp6v1a* or control, representative of two experiments.

(F) Average intramacrophage burden at 40 hpi with 60 Mm into the CV comparing larvae treated with DMSO or 50 nM Baf following infection, or injected with a morpholino targeting *tnfr1* at 0 dpf, which was used as a positive control, representative of three experiments.

(G) Bacterial burden measured at 3 dpi following infection with wild-type Mm, *marP*::Tn, *marP*::Tn transformed with a plasmid containing Mtb *marP*, or *marP*::Tn transformed with a plasmid containing Mtb *marP* with a mutation in the active site serine (S343A), representative of two experiments.

(H) Bacterial burden at 1 dpi following infection with wild-type or *marP*::Tn Mm and treatment with either DMSO or 50 nM Baf, representative of three experiments. The fold increase in burden following treatment with Baf is shown for wild-type and *marP*::Tn.

(I) Fold increase in bacterial burden during infection with wild-type or *marP*::Tn Mm following treatment with 50nM Baf. Shown is the average of four experiments as in (H) ± SEM.

(J) Bacterial burden measured at 0, 3, and 5 dpi following infection with 250 wild-type or *ptpA*::Tn Mm with mean and 95% CI shown, representative of three experiments.

(K) Bacterial burden at 0, 3, and 5 dpi following infection with wild-type or *marP*::Tn Mm with mean and 95% CI shown, representative of three experiments. Significance tested using two-tailed unpaired t test (B, D, E, and I–K) or one-way ANOVA with Tukey's post-test (F–H). Each point in (B) and (D)–(H) represents one larva. See also Figure S3.

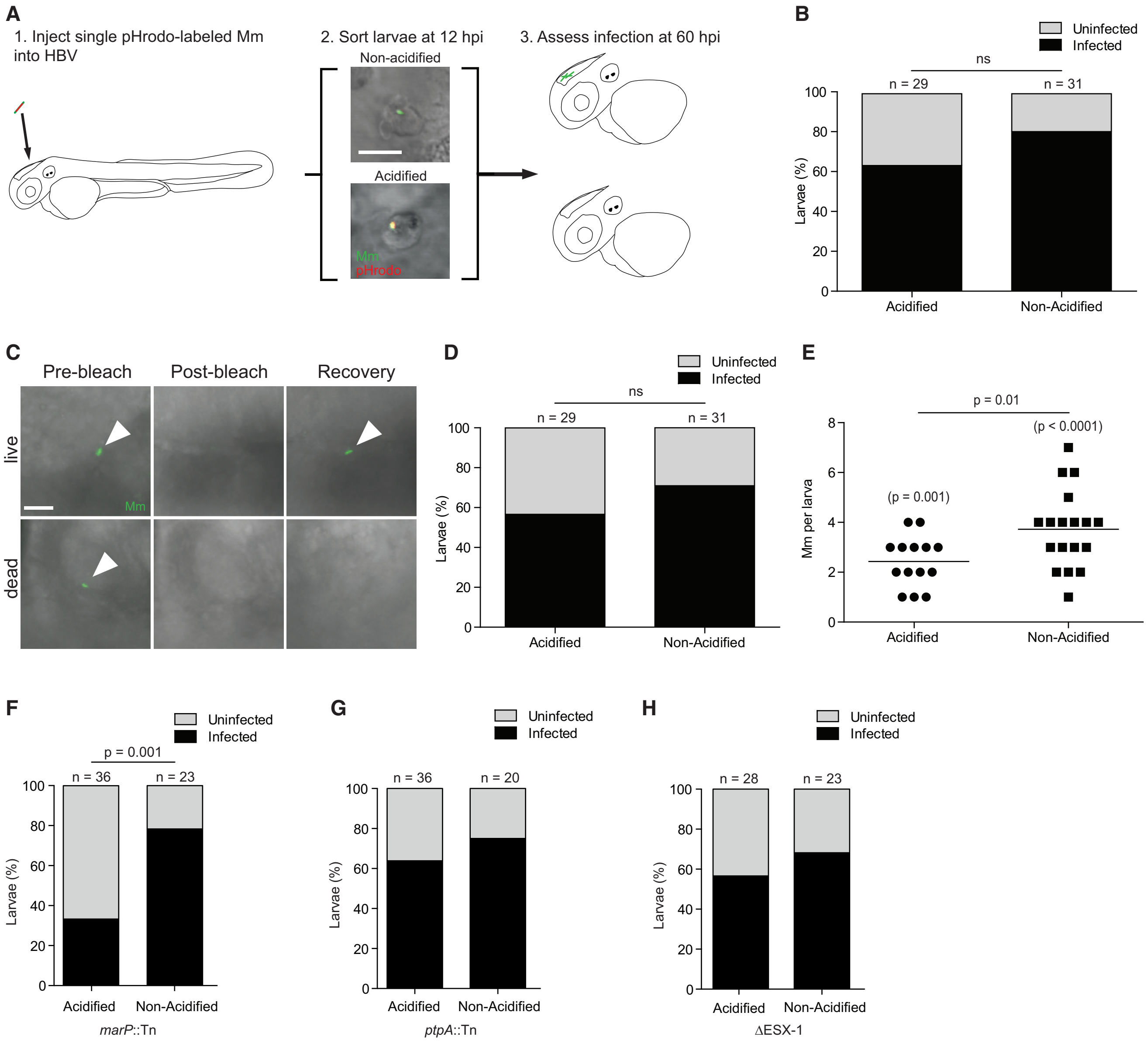

Figure 4. Lysosomal Trafficking Fails to Eradicate Mm Infection

(A) Cartoon diagram of infectivity experiment. Scale bar, 10 μm.

(B) Percent of zebrafish larvae still infected at 60 hpi following infection in the HBV with single pHrodo-labeled Mm and sorting into acidified/non-acidified groups, representative of three experiments.

(C) Photobleaching assay to discern live from killed bacteria at the end of the infectivity assay showing a live bacterium that recovers fluorescence and a dead one that does not. Scale bar, 5 μm.

(D) Data in (B) amended so that larvae with only non-recovering bacteria are placed into the uninfected category, representative of three experiments.

(E) Bacterial numbers at the end of the infectivity experiment, showing only bacteria that recovered after photobleaching, representative of three experiments. p values in parentheses reflect the statistical significance of comparing each larva in that group to the starting bacterial number (1 in each fish) in a Wilcoxon matched-pair signed rank test.

(F–H) Percentage of zebrafish larvae still infected at 60 hpi which contained pHrodo-labeled *marP*::Tn (F), *ptpA*::Tn (G), and ΔESX-1 (H) Mm separated by whether the bacteria were pHrodo-positive (lysosomal) or pHrodo-negative (phagosomal) at sorting, representative of two (ΔESX-1) or three (*marP*::Tn, *ptpA*::Tn) experiments. Significance tested using two-tailed unpaired t test (E) or Fisher's exact test (B, D, and F–H). Each point in (E) represents one larva. See also Figure S4.

bacteria, nor does it induce bacteriostasis. Mycobacteria continue to grow in both compartments, albeit more slowly in phagolysosomes. These in vivo findings differ from those in cultured macrophages, where lysosomal fusion was induced by Fc receptor-dependent phagocytosis and where decreased growth of the lysosomal bacteria was not observed (Armstrong and Hart, 1975). Our findings also differ from prior findings in vitro that while mycobacteria can survive transiently at low

pH, they fail to replicate under these conditions (Asrat et al., 2014; Behar and Baehrecke, 2015; Kasper et al., 2015; MacMicking, 2008; Tan and Russell, 2015). The ability of in vivo macrophage phagolysosomes to limit mycobacterial growth to some extent may reflect the presence of lysosomally activated immune determinants such as nitric oxide (Vandal et al., 2009). This in turn suggests that, ultimately, lysosomal trafficking may serve as a host-protective mechanism. However, its efficacy may be limited later in infection, as supported by previous work showing that morphologically intact Mm were present in frog granuloma macrophage lysosomes in unchanging and substantial numbers throughout a 17–52 week observation period when overall infection burdens were increasing (Bouley et al., 2001).

Prior work has shown that MarP is a virulence determinant in the mouse model of TB and that it is required for acid tolerance in vitro (Vandal et al., 2008). However, the link between MarP's role in acid tolerance and the attenuation of the *marP* mutant has remained tenuous because it has not been clear whether the mutant's attenuation is due to its acid intolerance or due to other effects such as sensitivity to oxidative stress or to cell wall insults (Ehrt et al., 2015; Stallings and Glickman, 2010). Our work firmly links the acid tolerance conferred by mycobacterial MarP to its function as a virulence determinant during the early stages of infection. Importantly, we show that MarP specifically enables the establishment of infection by mycobacteria that have been consigned to lysosomes. This likely represents a critical determinant of the evolutionary survival of Mtb; human TB is thought to begin with the deposition of one to three bacteria into the lung alveolus (Bates et al., 1965; Cambier et al., 2014a), and the initial interaction between these bacteria and the host macrophage is thought to determine whether the infection is cleared or progresses (Verrall et al., 2014). MarP is widely conserved across mycobacterial species, which have been reported to survive (and even be enriched) in environments as hostile as volcanic rock at pH 1 (Walker et al., 2005) (Figure S4). Thus, acid tolerance likely evolved as a survival mechanism well before mycobacteria encountered macrophages or free-living amebae and joins the catalog of determinants for environmental survival that have been repurposed for host survival by pathogenic mycobacteria (Cambier et al., 2014a).

EXPERIMENTAL PROCEDURES

Bacterial Strains and Methods

Wild-type Mm (strain M, ATCC #BAA-535) expressing tdTomato or mWasabi under the constitutive promoter *msp12* was used for measurement of total and intracellular bacterial burdens. *mmar_5159* and *mmar_3309* mutants were isolated from a library of Mm transposon mutants (C.L.C., unpublished data). See also Supplemental Experimental Procedures.

Mouse Husbandry and Mtb Infection

Mouse husbandry and experiments were conducted in accordance with an animal study proposal approved by the Center for Infectious Disease Research Animal Care and Use Committee. C57BL/6 mice purchased from Jackson Laboratories or maintained in house were infected with ~200 CFU of aerosolized Mtb strain H37Rv expressing mCherry in a Glas-Col infection chamber (Glas-Col, Terre Haute, IN). Two mice from each infection were sacrificed and lung homogenates plated to determine the deposition of Mtb.

Zebrafish Husbandry and Mm Infections in Zebrafish

Zebrafish husbandry and experiments were in compliance with guidelines from the UK Home Office and the U.S. National Institutes of Health and approved by the University of Washington Institutional Animal Care and Use Committee. Transgenic lines were maintained as outcrosses to AB. Larvae were infected at 48–72 hr post-fertilization via CV or HBV injection using thawed single-cell suspensions with 75–200 bacteria delivered during infection unless noted otherwise. Fish containing single bacteria were identified 3 hpi by confocal microscopy and were again scored at 12 hpi for pHrodo sorting using cutoff parameters as in Figure S2A. See also Supplemental Experimental Procedures.

Statistical Analyses and Image Analysis

Images were analyzed using Imaris 7.7–8.2 (Bitplane). The Venn diagram was generated using the Pan-Omics Research Venn Diagram Plotter (http://omics.pnl.gov). Statistical analyses were performed using Prism 6 (GraphPad) (not significant [ns], $p \geq 0.05$; * $p < 0.05$; ** $p < 0.01$; *** $p < 0.001$; **** $p < 0.0001$).

SUPPLEMENTAL INFORMATION

Supplemental Information includes four figures, one table, and Supplemental Experimental Procedures and can be found with this article at http://dx.doi.org/10.1016/j.chom.2016.07.007.

AUTHOR CONTRIBUTIONS

S.L., K.N.A., R.D.B., K.B.U., and L.R. conceived and designed experiments and analyzed the data. C.L.C. generated Mm mutants. S.L. and L.R. wrote the paper with input from C.L.C. and R.D.B. The figures were prepared by S.L.

ACKNOWLEDGMENTS

We thank S. Ehrt for Mtb MarP plasmids; C.J. Cambier for advice on infectivity experiments; A. Pagán and P. Edelstein for manuscript review; N. Goodwin, R. Keeble, and J. Cameron for fish facility management; K. Takaki for help with figure preparation; S. Renshaw for suggesting pHrodo and M. Troll for advice on using it and analyzing the data; and K. Minch for BSL3 technical assistance. This work was supported by the National Institutes of Health (R37AI054503 to L.R., R01 AI076327 to K.B.U., 5T32HD007233 to K.N.A., 5F30HL110455 to R.D.B.), the Wellcome Trust (L.R.), and the National Institute of Health Research Cambridge Biomedical Research Centre (L.R.).

Received: May 10, 2016
Revised: June 20, 2016
Accepted: July 19, 2016
Published: August 10, 2016

REFERENCES

Aggad, D., Stein, C., Sieger, D., Mazel, M., Boudinot, P., Herbomel, P., Levraud, J.-P., Lutfalla, G., and Leptin, M. (2010). In vivo analysis of Ifn-g1 and Ifn-g2 signalling in zebrafish. J. Immunol. *185*, 6774–6782.

Armstrong, J.A., and Hart, P.D. (1971). Response of cultured macrophages to Mycobacterium tuberculosis, with observations on fusion of lysosomes with phagosomes. J. Exp. Med. *134*, 713–740.

Armstrong, J.A., and Hart, P.D. (1975). Phagosome-lysosome interactions in cultured macrophages infected with virulent tubercle bacilli. Reversal of the usual nonfusion pattern and observations on bacterial survival. J. Exp. Med. *142*, 1–16.

Asrat, S., de Jesús, D.A., Hempstead, A.D., Ramabhadran, V., and Isberg, R.R. (2014). Bacterial pathogen manipulation of host membrane trafficking. Annu. Rev. Cell Dev. Biol. *30*, 79–109.

Bach, H., Papavinasasundaram, K.G., Wong, D., Hmama, Z., and Av-Gay, Y. (2008). Mycobacterium tuberculosis virulence is mediated by PtpA dephosphorylation of human vacuolar protein sorting 33B. Cell Host Microbe *3*, 316–322.

Baldridge, M.T., King, K.Y., Boles, N.C., Weksberg, D.C., and Goodell, M.A. (2010). Quiescent haematopoietic stem cells are activated by IFN-gamma in response to chronic infection. Nature *465*, 793–797.

Barker, L.P., George, K.M., Falkow, S., and Small, P.L. (1997). Differential trafficking of live and dead Mycobacterium marinum organisms in macrophages. Infect. Immun. *65*, 1497–1504.

Bates, J.H., Potts, W.E., and Lewis, M. (1965). Epidemiology of Primary Tuberculosis in an Industrial School. N. Engl. J. Med. *272*, 714–717.

Behar, S.M., and Baehrecke, E.H. (2015). Tuberculosis: Autophagy is not the answer. Nature *528*, 482–483.

Bouley, D.M., Ghori, N., Mercer, K.L., Falkow, S., and Ramakrishnan, L. (2001). Dynamic nature of host-pathogen interactions in Mycobacterium marinum granulomas. Infect. Immun. *69*, 7820–7831.

Cambier, C.J., Falkow, S., and Ramakrishnan, L. (2014a). Host evasion and exploitation schemes of Mycobacterium tuberculosis. Cell *159*, 1497–1509.

Cambier, C.J., Takaki, K.K., Larson, R.P., Hernandez, R.E., Tobin, D.M., Urdahl, K.B., Cosma, C.L., and Ramakrishnan, L. (2014b). Mycobacteria manipulate macrophage recruitment through coordinated use of membrane lipids. Nature *505*, 218–222.

Clay, H., Volkman, H.E., and Ramakrishnan, L. (2008). Tumor necrosis factor signaling mediates resistance to mycobacteria by inhibiting bacterial growth and macrophage death. Immunity *29*, 283–294.

Clements, W.K., and Traver, D. (2013). Signalling pathways that control vertebrate haematopoietic stem cell specification. Nat. Rev. Immunol. *13*, 336–348.

Cosma, C.L., Sherman, D.R., and Ramakrishnan, L. (2003). The secret lives of the pathogenic mycobacteria. Annu. Rev. Microbiol. *57*, 641–676.

Ehrt, S., Rhee, K., and Schnappinger, D. (2015). Mycobacterial genes essential for the pathogen's survival in the host. Immunol. Rev. *264*, 319–326.

Flynn, J.L., and Chan, J. (2001). Immunology of tuberculosis. Annu. Rev. Immunol. *19*, 93–129.

Gomes, M.S., Paul, S., Moreira, A.L., Appelberg, R., Rabinovitch, M., and Kaplan, G. (1999). Survival of Mycobacterium avium and Mycobacterium tuberculosis in acidified vacuoles of murine macrophages. Infect. Immun. *67*, 3199–3206.

Grundner, C., Cox, J.S., and Alber, T. (2008). Protein tyrosine phosphatase PtpA is not required for Mycobacterium tuberculosis growth in mice. FEMS Microbiol. Lett. *287*, 181–184.

Horng, J.L., Lin, L.Y., Huang, C.J., Katoh, F., Kaneko, T., and Hwang, P.P. (2007). Knockdown of V-ATPase subunit A (atp6v1a) impairs acid secretion and ion balance in zebrafish (Danio rerio). Am. J. Physiol. Regul. Integr. Comp. Physiol. *292*, R2068–R2076.

Huynh, K.K., and Grinstein, S. (2007). Regulation of vacuolar pH and its modulation by some microbial species. Microbiol. Mol. Biol. Rev. *71*, 452–462.

Kasper, D.L., Fauci, A.S., Hauser, S.L., Longo, D.L., Jameson, J.L., Loscalzo, J., and Harrison, T.R.; McGraw-Hill Companies (2015). Harrison's principles of internal medicine. In AccessMedicine (New York: McGraw Hill Education), p. 1.

Khader, S.A., Bell, G.K., Pearl, J.E., Fountain, J.J., Rangel-Moreno, J., Cilley, G.E., Shen, F., Eaton, S.M., Gaffen, S.L., Swain, S.L., et al. (2007). IL-23 and IL-17 in the establishment of protective pulmonary CD4+ T cell responses after vaccination and during Mycobacterium tuberculosis challenge. Nat. Immunol. *8*, 369–377.

MacGurn, J.A., and Cox, J.S. (2007). A genetic screen for Mycobacterium tuberculosis mutants defective for phagosome maturation arrest identifies components of the ESX-1 secretion system. Infect. Immun. *75*, 2668–2678.

MacMicking, J.D. (2008). M. tuberculosis passes the litmus test. Nat. Med. *14*, 809–810.

Mwandumba, H.C., Russell, D.G., Nyirenda, M.H., Anderson, J., White, S.A., Molyneux, M.E., and Squire, S.B. (2004). Mycobacterium tuberculosis resides in nonacidified vacuoles in endocytically competent alveolar macrophages from patients with tuberculosis and HIV infection. J. Immunol. *172*, 4592–4598.

Nunes-Alves, C., Booty, M.G., Carpenter, S.M., Jayaraman, P., Rothchild, A.C., and Behar, S.M. (2014). In search of a new paradigm for protective immunity to TB. Nat. Rev. Microbiol. *12*, 289–299.

Peri, F., and Nüsslein-Volhard, C. (2008). Live imaging of neuronal degradation by microglia reveals a role for v0-ATPase a1 in phagosomal fusion in vivo. Cell *133*, 916–927.

Renshaw, S.A., and Trede, N.S. (2012). A model 450 million years in the making: zebrafish and vertebrate immunity. Dis. Model. Mech. *5*, 38–47.

Schaible, U.E., Sturgill-Koszycki, S., Schlesinger, P.H., and Russell, D.G. (1998). Cytokine activation leads to acidification and increases maturation of Mycobacterium avium-containing phagosomes in murine macrophages. J. Immunol. *160*, 1290–1296.

Sieger, D., Stein, C., Neifer, D., van der Sar, A.M., and Leptin, M. (2009). The role of gamma interferon in innate immunity in the zebrafish embryo. Dis. Model. Mech. *2*, 571–581.

Small, J.L., O'Donoghue, A.J., Boritsch, E.C., Tsodikov, O.V., Knudsen, G.M., Vandal, O., Craik, C.S., and Ehrt, S. (2013). Substrate specificity of MarP, a periplasmic protease required for resistance to acid and oxidative stress in Mycobacterium tuberculosis. J. Biol. Chem. *288*, 12489–12499.

Stallings, C.L., and Glickman, M.S. (2010). Is Mycobacterium tuberculosis stressed out? A critical assessment of the genetic evidence. Microbes Infect. *12*, 1091–1101.

Tan, S., and Russell, D.G. (2015). Trans-species communication in the Mycobacterium tuberculosis-infected macrophage. Immunol. Rev. *264*, 233–248.

Urdahl, K.B. (2014). Understanding and overcoming the barriers to T cell-mediated immunity against tuberculosis. Semin. Immunol. *26*, 578–587.

Vandal, O.H., Pierini, L.M., Schnappinger, D., Nathan, C.F., and Ehrt, S. (2008). A membrane protein preserves intrabacterial pH in intraphagosomal Mycobacterium tuberculosis. Nat. Med. *14*, 849–854.

Vandal, O.H., Nathan, C.F., and Ehrt, S. (2009). Acid resistance in Mycobacterium tuberculosis. J. Bacteriol. *191*, 4714–4721.

Verrall, A.J., Netea, M.G., Alisjahbana, B., Hill, P.C., and van Crevel, R. (2014). Early clearance of Mycobacterium tuberculosis: a new frontier in prevention. Immunology *141*, 506–513.

Via, L.E., Fratti, R.A., McFalone, M., Pagan-Ramos, E., Deretic, D., and Deretic, V. (1998). Effects of cytokines on mycobacterial phagosome maturation. J. Cell Sci. *111*, 897–905.

Walker, J.J., Spear, J.R., and Pace, N.R. (2005). Geobiology of a microbial endolithic community in the Yellowstone geothermal environment. Nature *434*, 1011–1014.

Wolf, A.J., Linas, B., Trevejo-Nuñez, G.J., Kincaid, E., Tamura, T., Takatsu, K., and Ernst, J.D. (2007). Mycobacterium tuberculosis infects dendritic cells with high frequency and impairs their function in vivo. J. Immunol. *179*, 2509–2519.

Cell Host & Microbe, Volume *20*

Supplemental Information

Mycobacterial Acid Tolerance Enables Phagolysosomal Survival and Establishment of Tuberculous Infection In Vivo

Steven Levitte, Kristin N. Adams, Russell D. Berg, Christine L. Cosma, Kevin B. Urdahl, and Lalita Ramakrishnan

Supplemental Figures and Tables

Figure S1

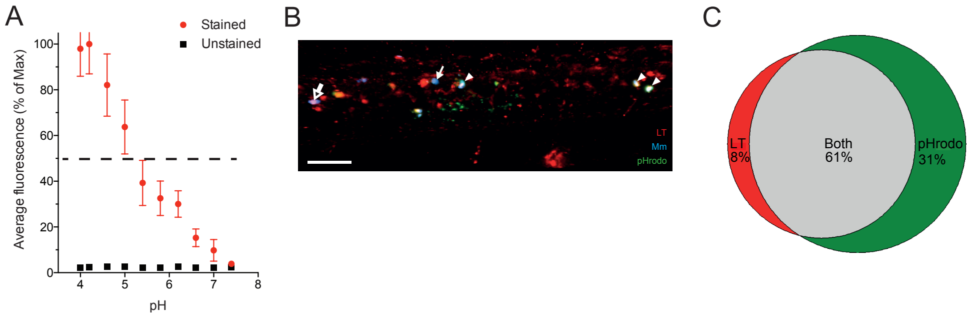

Figure S1. pHrodo efficiently labels acidified *M. marinum* (Mm), related to Figure 2. (A) Intensity of pHrodo fluorescence in stained Mm at various pH levels. Bacteria were pre-labeled with pHrodo, and then incubated in phosphate-citrate buffer at the indicated pH. The stained bacteria and unstained controls were imaged using the same confocal parameters as those used in zebrafish larvae. 3D surfaces were created from these images and pHrodo intensity was measured within each bacterium and intensity was plotted as a percentage of the maximum average value (pH 4.2). Shown is the average of at least 300 individual bacteria +/- one standard deviation. The dotted line represents the intensity cutoff used to assess pHrodo in vivo. (B) Confocal image of larva infected in the CV with pHrodo-labeled Mm, then stained with LysoTracker at 24hpi. White arrowheads denote bacteria that label with both LysoTracker and pHrodo, white arrow denotes a bacterium that does not label with either marker, and black arrow denotes a bacterium that labels with LysoTracker but not with pHrodo. Scale bar, 50µm. (C) Quantitative Venn diagram showing the distribution of Mm that were positive for either LysoTracker (LT), pHrodo or both in larvae infected in the CV with pHrodo-labeled Mm and then stained with LysoTracker at 24hpi with percentages noted, representative of two experiments.

Figure S2

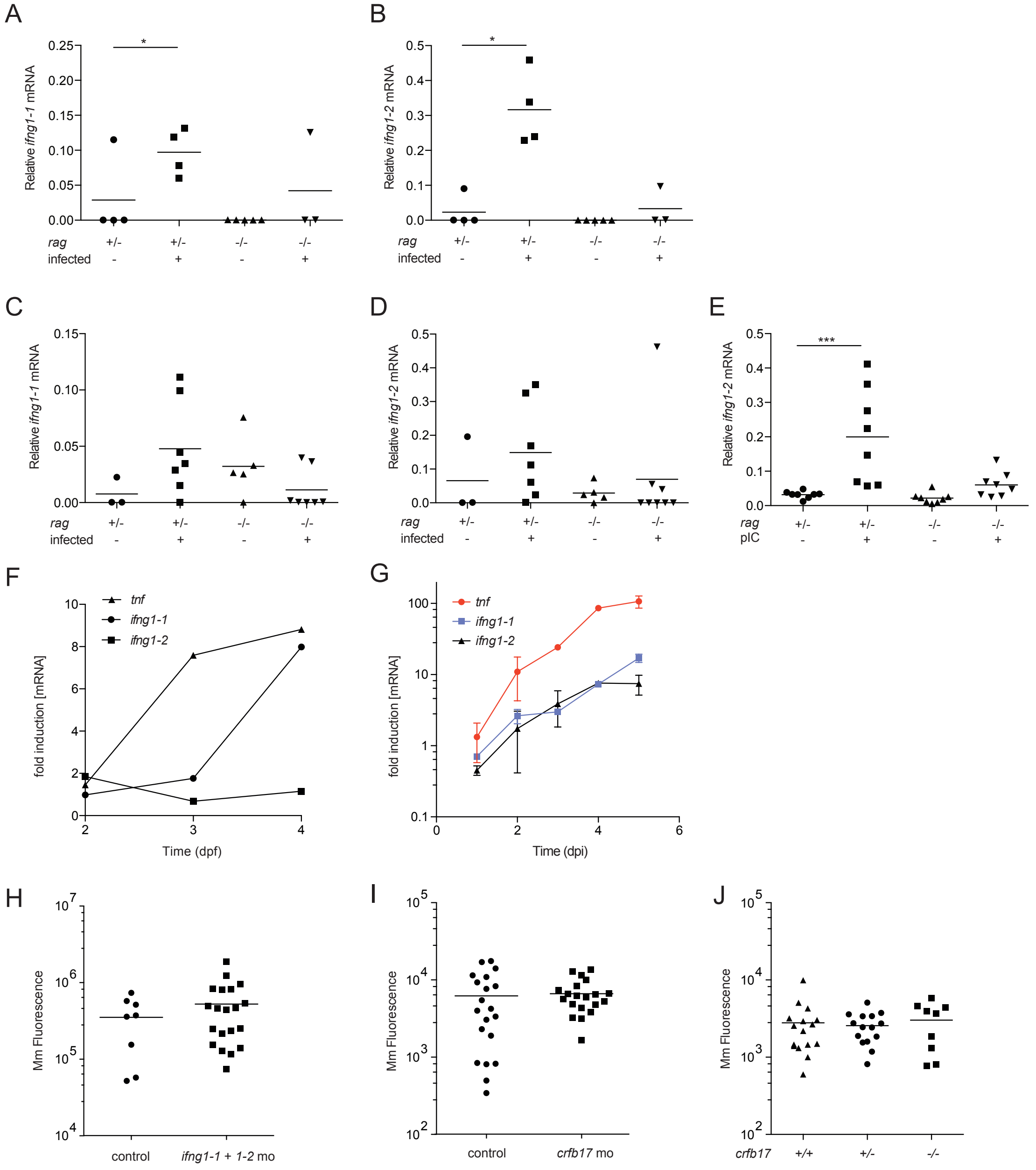

Figure S2. IFNγ induction does not occur in zebrafish larvae younger than 4dpf, related to Figure 2. (A, B) Real-time qPCR of *ifng1-1* (A) and *ifng1-2* (B) mRNA expression relative to *b-actin* in adult zebrafish at 6 weeks post-intraperitoneal infection with 20 CFU Mm in *rag* heterozygote and mutant adults. (C, D) Real-time qPCR of *ifng1-1* (C) and *ifng1-2* (D) mRNA expression relative to *b-actin* in adult zebrafish at 2 weeks post-intraperitoneal infection with 500 CFU Mm in *rag* heterozygote and mutant adults. (E) Real-time qPCR of *ifng1-2* mRNA expression relative to *b-actin* in adult zebrafish four hours after intraperitoneal injection with poly(I:C). (F) Real-time qPCR of *ifng1-1*, *ifng1-2* and *tnf* mRNA expression relative to *b-actin* in larval zebrafish four hours after intravenous injection with poly(I:C). (G)

Real-time qPCR of selected gene products following CV infection of 2dpf larvae with 275 CFU *M. marinum*. Shown for each point is an average of three experiments +/- SEM. (H) Bacterial burden measured at 4dpi following infection with 200 Mm in larvae treated at 0dpi with combined morpholinos targeting *ifng1-1* and *ifng1-2* or control. (I) Bacterial burden measured at 6dpi following infection with 200 Mm in larvae treated at 0dpi with a morpholino targeting *crfb17* or control. (J) Bacterial burden measured at 5dpi following infection with 250 Mm larvae from an incross of *crfb17* heterozygous (+/-) parents. Significance tested using ANOVA with Kruskal-Wallis multiple comparisons test. TNF induction was used as an independent indicator that adults and larvae were successfully infected. Each point in A-E represents one adult with the same adults tested in A-B and C-D. Each point in H-J represents one larva. Values in (F) and (G) represent pooled RNA from 30 larvae at each time point.

Figure S3

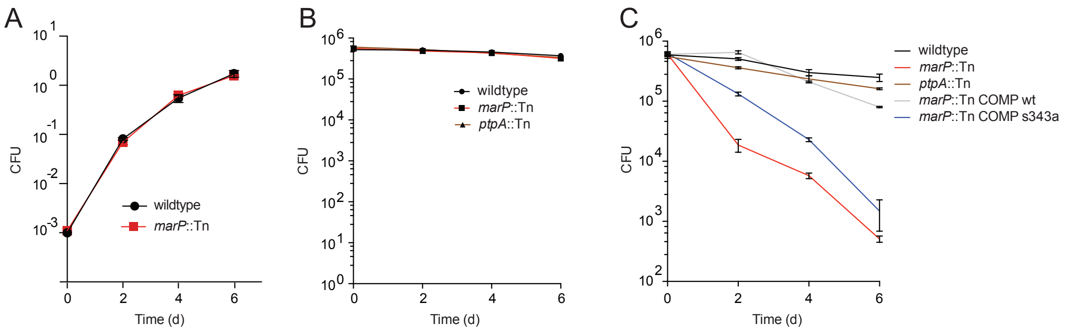

D

Strain	MIC (µg/mL)	
	Rif	Ery
wildtype	0.16	64
ΔESX-1	0.16	64
Δ*erp*	0.04	2
marP::Tn	0.02	2
ptpA::Tn	0.16	64

Figure S3. *marP*::Tn is unable to tolerate acidic environments and lipophilic antibiotics, related to Figure 3. (A) OD600 measurements of wild-type and *marP*::Tn in 7H9 media at neutral pH over 6 days of growth. (B) Wild-type, *marP*::Tn and *ptpA*::Tn bacteria were incubated for 6 days in phosphate-citrate buffer at pH 7 with CFU measurements taken every 2 days. (C) Wild-type, *marP*::Tn, *ptpA*::Tn, *marP*::Tn complemented with *marP* from Mtb and *marP*::Tn complemented with the *marP* S343A mutant were incubated in phosphate-citrate buffer at pH 4.5 for 6 days with CFU measurements taken every 2 days. (D) MIC measurements of hydrophobic antibiotics in wild-type, ΔESX-1, Δ*erp*, *marP*::Tn and *ptpA*::Tn. MICs were measured as described in Experimental Procedures and was determined as the minimum concentration that prevented the appearance of turbidity in culture. Rif, rifampin; Ery, erythromycin. Values in A, B, and C represent the mean +/- SD.

Figure S4

```
M. tuberculosis              PVTRDVYTIRADVEQGDSGGPLIDLNGQVLGVVFGAAID----DAETGFVLTA
M. bovis (99% identity)      PVTRDVYTIRADVEQGDSGGPLIDLNGQVLGVVFGAAVD----DAETGFVLTA
M. avium (86% identity)      PVTRDVYTIRASVEQGNSGGPLIDLNGQVLGVVFGAAVD----DPDTGFVLTA
M. elephantis (67% identity) TVEREVYTIRGTVRQGNSGGPMIDRDGNVLGVVFGAAVD----DADTGFVLTA
M. smegmatis (66% identity)  TVTREVYTVRGTVRQGNSGGPMINRAGKVLGVVFGAAVD----DVDTGFVLTA
M. marinum (86% identity)    TLNGLIQV-DAAIAPGDSGGPIVNNMGQVVGMNTAASDNFQMSGGGTGFAIPI
```

Figure S4. The serine protease encoded by MarP is highly conserved across mycobacterial species, related to Figure 4. Alignment of a section of MarP from Mtb (with active site serine S343 shown in red) with other species of mycobacteria. The overall identity between a given species and the entire gene from Mtb is reported in parentheses (alignments performed using LALIGN from NCBI database sequences).

Table S1. Prevalence of phagolysosomal trafficking of mycobacteria in vitro and in vivo

In vitro			
**Bacterium**	**Cell type**	**% phagosomes acidified (time)**	**Reference**
M. tuberculosis	Mouse peritoneal macrophages	36 (1 day)<br>23 (4 days)	Armstrong and Hart, 1971[7]
M. tuberculosis	Mouse peritoneal macrophages	23 (2 hours)	Hart *et al.,* 1972[8]
M. tuberculosis (immune rabbit serum treated)	Mouse peritoneal macrophages	79 (1 day)<br>68 (7 days)	Armstrong and Hart, 1975[9]
M. tuberculosis	Human primary mononuclear cells	25 (3 hours)<br>15 (22 hours)	Clemens and Horwitz 1995[10]
M. tuberculosis	Mouse bone marrow macrophages	10 (2 hours)	Pethe *et al*, 2004[11]
M. tuberculosis	Human macrophage cell line (THP-1)<br><br>THP-1 stimulated with IFNγ	10 (2 hours)<br><br>25 (2 hours)	Harris *et al,* 2008[12]
M. marinum	Mouse macrophage cell line (RAW)	21 (4 hours)<br>21 (8 hours)<br>27 (24 hours)	Barker *et al*, 1997[13]

In vivo			
**Bacterium**	**Host**	**% phagosomes acidified (time)**	**Reference**
M. tuberculosis	Mouse	30 (3 days)	Jayachandran *et al*., 2007[14]
M. tuberculosis	Human	30* (indeterminate)¢	Mwandumba *et al*., 2004[15]

Table S1. Related to Figures 1 and 2. Quantitation of lysosomal fusion of mycobacterial phagosomes as compiled from the literature. Note that in cases where bar graphs were shown for acidification instead of exact numbers, the extent was estimated to the nearest 5%.

*Estimated from Figure 6 of the paper

¢Patients presented with suspected pulmonary tuberculosis.

Supplemental Experimental Procedures

Bacterial Strains

Single-cell stocks were prepared as described for injection, and inocula were determined by injection onto selective 7H10 plates (Takaki et al., 2013). Heat-killed bacteria were prepared by incubating at 80°C for 20 minutes (Cambier et al., 2014). To make the transposon mutants, a transposon was created that contains an excisable hygromycin-resistance cassette to allow capture of transposon proximal sequences and this transposon was used to mutagenize wildtype Mm. The location of the transposon insertion in each gene was confirmed by sequencing prior to use. Wildtype Mm expressing EBFP2 under the *msp12* promoter was used as noted. All Mm strains were grown under hygromycin (Mediatech), streptomycin (Mediatech), or kanamycin (Mediatech) selection in Middlebrook's 7H9 medium (Difco) supplemented with glycerol, oleic acid, albumin, dextrose and Tween-80 (Takaki et al., 2013).

Mouse imaging

For ex vivo imaging studies, single cell lung preparations were made as previously described (Moguche et al., 2015). Infected (mCherry expressing) cells were isolated by FACS sorting with a FACSAria II (BD Biosciences, Franklin Lakes, NJ). Infected cells were then seeded onto 24-well glass-bottom plates coated with Cell-Tak adhesive (Corning) and incubated with 75nM LysoTracker Green DND-26 (Molecular Probes) for 30 minutes. Cells were washed and immediately imaged using an inverted Nikon microscope fitted with 20x and 40x objectives.

Zebrafish husbandry and injections

Larvae were maintained in fish water supplemented with *N*-phenylthiourea (PTU) from 1 day post-fertilization. The Tg(*mpeg1:YFP)*^{w200} line was used as previously described (Pagan et al., 2015). Larvae were randomly assigned to different experimental conditions, and were assigned after infection for all drug treatment studies. For the infectivity assays, larvae were infected in the HBV at 2.5dpf with 0.4 bacteria per injection. Heterozygous *rag1* mutant zebrafish were obtained from Artemis Pharmaceuticals (Köln, Germany) (Wienholds et al., 2002) and maintained as heterozygotes via outcrosses to the AB line. Carriers were incrossed to generate *rag1*-/- individuals. Genotype was determined at 3 to 6 months of age using DNA obtained from a tail clip procedure as described previously (Swaim et al., 2006) using a Taqman genotyping mix containing the following primers and probes: rag1_Forward 5'-CTCAGAGTCAGCAGACGAACTG-3' , rag1_Reverse 5'-GGTTTCCATGAAAGGCTTAGCAAAA-3', rag1_WT Reporter 5'-CCTTTGACTCGGTCACG-3', rag1_ Mutant Reporter 5'-CCTTTGACTCAGTCACG-3'. Adult intraperitoneal injections were conducted as previously described (Cosma et al., 2006). Appropriate dilutions of single cell *M. marinum* stocks were used for infection so that 5μl would contain approximately 500 CFU (acute infection) or 20 CFU (chronic infection). Inoculum was confirmed with plate counts (Cosma et al., 2006). Crfb17 IFNγ receptor mutants (sa1747 Sanger Zebrafish Resource) was confirmed with sequencing and then High Resolution Melt Analysis using the same primers: crfb17_HRMF: 5'- TGTCTCGAGCAGCGTATAATG-3', crfb17_HRMR: 5'- GCTGCTTCCATGTTGATTGA – 3'. Intramacrophage burdens were enumerated as described (Takaki et al., 2013). Poly (I:C) - Polyinosinic-polycytidylic acid (poly I:C) (Sigma-Aldrich P1530) was dissolved in PBS buffer to a concentration of 5mg/mL and sterile filtered. For adult injections, 5μL of this solution vs 5μL PBS was injected intraperitoneally. For larval injections, 2dpf larvae were injected with approximately 10nL of poly (I:C) 5mg/mL solution in the caudal vein.

Microscopy

Fluorescence microscopy was performed as described (Pagan et al., 2015; Takaki et al., 2013). Quantification of bacterial burdens was performed using an inverted Nikon TiE microscope fitted with 4x and 10x objectives. For confocal microscopy, larvae were anesthetized in fish water with 0.025% Tricaine and embedded either in 1.5% low melting-point agarose or 2% hydroxymethylcellulose on optical bottom plates (MatTek). Confocal microscopy was done using a Nikon TiE microscope with 20x Plan Apo 0.75NA objective with A1 confocal system using a galvano scanner to generate 20-60μm stacks with 1-1.5μm vertical spacing. Photobleaching was performed using the 488nm laser on the confocal microscope with the minimal power and dwell time required to eliminate detection of bacterial fluorescence in the green

channel. Larvae were incubated at 28°C for 12 hours prior to assessing for fluorescence recovery. Data were acquired using NIS Elements version 4.4. Microscope scoring of staining, and bacterial enumeration, were performed in a blinded manner whenever possible.

Staining

LysoTracker Red DND-99 dye (DMSO solution) (Molecular Probes) was diluted 1:25 in PBS prior to injection of 5nL into the HBV or CV of larvae, which were then incubated for 1hr prior to imaging. MagicRed-Cathepsin (Immunochemistry Technologies) was resuspended at the concentration suggested by the manufacturer in DMSO, diluted 1:4 in 1xPBS prior to injection into the HBV or CV of larvae which were incubated for 1.5hr prior to imaging. pHrodo Green STP Ester and pHrodo Red succinimidyl ester (Molecular Probes) https://www.thermofisher.com/uk/en/home/references/molecular-probes-the-handbook/ph-indicators/probes-useful-at-acidic-ph.html#head4 were resuspended in DMSO according to manufacturer instructions and stored in the dark at -20°C until use. Thawed single cell preparations of *M. marinum* were resuspended in 200μL 1xPBS and incubated with 1μL pHrodo Green or Red for 30 min at 30°C. The bacteria were washed once with 1xPBS and resuspended in PBS pH 7.4 prior to injection into the zebrafish, or resuspended in phosphate-citrate buffer of appropriate pH for in vitro studies. For single-cell infections, the bacteria were confirmed to be pHrodo-negative at the time of dose confirmation.

Morpholinos

Morpholino oligonucleotides (mo) were designed and synthesized by Gene Tools (Philomath, OR). Morpholinos were diluted in 1x Buffer Tango (ThermoFisher) 2% phenol red sodium salt solution (Sigma) and injected 1nL into the yolk of 1 cell-stage embryos. *atp6v1a* translation blocking morpholino sequence: ATCCATCTTGTGTGTTAGAAAACTG as described (Horng et al., 2007). *tnfr1* morpholino was used as described (Roca and Ramakrishnan, 2013). *tnfr1* morpholino targeting the exon 5 / intron 6 boundary: CTGCATTGTGACTTACTTATCGCAC. *crfb17* splice blocking morpholino (GQ901865) TTAAACTAAATCGCCTTACCTTGTG, *ifng1* (NM_001020793) AAAAGAATACTGACCAGCATAGATG and *ifng2* (NM_212864) TGAAGGCGTTCGCTAAAGTTAGAGT were used as described (Aggad et al., 2010).

Drug treatments

A stock of Bafilomycin A1 (Cambridge Bioscience) was dissolved in DMSO prior to use. Larvae were treated with Baf at 50nM in 0.5% DMSO via soaking at 2dpf following infection and were assessed at 24-40 hpi.

RNA isolation and quantitative real-time PCR (qRT-PCR)

Adult fish were euthanized according to approved Animal Use Protocols. Fish were then flash-frozen with liquid nitrogen and homogenized using a mortar and pestle in 5ml Trizol reagent (Life Technologies). A 1mL aliquot was then further processed according to the manufacturer's protocol. Larval zebrafish were euthanized according to approved Animal Use Protocols. Total RNA from batches of ~30 embryos per biological replicate was isolated with TRIzol Reagent (Life Technologies) and used to synthesize cDNA with Superscript II reverse transcriptase and oligo-dT primers (Invitrogen). Quantitative RT-PCR was performed as previously described (Clay et al., 2007) with SYBR green PCR Master Mix (Applied Biosystems) on an ABI Prism 7300 Real Time PCR System (Applied Biosystems). Each biological replicate was run in triplicate, and average values were plotted. Data were normalized to *b-actin* for ΔΔCt analysis. The primers used in this study were: ifng1-1 Forward = 5'-ATTCCTGCCTCAAAATGGTG-3', ifng1-1 Reverse 5' – TTTTCCAACCCAATCCTTTG-3', ifng1-2 Forward 5'- CTATGGGCGATCAAGGAAAA-3', ifng1-2 Reverse 5'= CTTTAGCCTGCCGTCTCTTG-3', bactin Forward 5'-ACCTCATGAAGATCCTGACC-3', bactin Reverse 5'-TGCTAATCCACATCTGCTGG-3', tnf Forward 5'-AGGCAATTTCACTTCCAAGG-3', tnf Reverse 5'-CAAGCCACCTGAAGAAAAGG-3'

MIC assays

MICs for each strain were determined in 7H9 broth. A suspension of single bacteria was inoculated into 7H9 ($2x10^4$ ml^{-1}) containing antibiotics at different concentration and cultured for 6 days before scoring for

turbidity with each concentration tested in triplicate. The MIC was defined as the minimal concentration at which turbidity was not observed.

Supplemental References

Aggad, D., Stein, C., Sieger, D., Mazel, M., Boudinot, P., Herbomel, P., Levraud, J.-P., Lutfalla, G., and Leptin, M. (2010). In Vivo Analysis of Ifn-g1 and Ifn-g2 Signalling in Zebrafish. Journal of Immunology *185*.

Cambier, C.J., Takaki, K.K., Larson, R.P., Hernandez, R.E., Tobin, D.M., Urdahl, K.B., Cosma, C.L., and Ramakrishnan, L. (2014). Mycobacteria manipulate macrophage recruitment through coordinated use of membrane lipids. Nature *505*, 218-222.

Clay, H., Davis, J.M., Beery, D., Huttenlocher, A., Lyons, S.E., and Ramakrishnan, L. (2007). Dichotomous role of the macrophage in early Mycobacterium marinum infection of the zebrafish. Cell Host and Microbe *2*, 29-39.

Cosma, C.L., Swaim, L.E., Volkman, H., Ramakrishnan, L., and Davis, J.M. (2006). Zebrafish and frog models of Mycobacterium marinum infection. Curr Protoc Microbiol *Chapter 10*, Unit 10B.12.

Horng, J.L., Lin, L.Y., Huang, C.J., Katoh, F., Kaneko, T., and Hwang, P.P. (2007). Knockdown of V-ATPase subunit A (atp6v1a) impairs acid secretion and ion balance in zebrafish (Danio rerio). Am J Physiol Regul Integr Comp Physiol *292*, R2068-2076.

Moguche, A.O., Shafiani, S., Clemons, C., Larson, R.P., Dinh, C., Higdon, L.E., Cambier, C.J., Sissons, J.R., Gallegos, A.M., Fink, P.J*., et al*. (2015). ICOS and Bcl6-dependent pathways maintain a CD4 T cell population with memory-like properties during tuberculosis. The Journal of experimental medicine *212*, 715-728.

Pagan, A.J., Yang, C.T., Cameron, J., Swaim, L.E., Ellett, F., Lieschke, G.J., and Ramakrishnan, L. (2015). Myeloid Growth Factors Promote Resistance to Mycobacterial Infection by Curtailing Granuloma Necrosis through Macrophage Replenishment. Cell Host Microbe *18*, 15-26.

Roca, F.J., and Ramakrishnan, L. (2013). TNF dually mediates resistance and susceptibility to mycobacteria via mitochondrial reactive oxygen species. Cell *153*, 521-534.

Swaim, L.E., Connolly, L.E., Volkman, H.E., Humbert, O., Born, D.E., and Ramakrishnan, L. (2006). Mycobacterium marinum infection of adult zebrafish produces caseating granulomatous tuberculosis and is moderated by adaptive immunity. Infect Immun *74*, 6108-6117.

Takaki, K., Davis, J.M., Winglee, K., and Ramakrishnan, L. (2013). Evaluation of the pathogenesis and treatment of Mycobacterium marinum infection in zebrafish. Nat Protoc *8*, 1114-1124.

Wienholds, E., Schulte-Merker, S., Walderich, B., and Plasterk, R.H. (2002). Target-selected inactivation of the zebrafish rag1 gene. Science *297*, 99-102.
